# Chromosome‐Level Genome Assembly of the Leafcutter Bee *Megachile rotundata* Reveals Its Ecological Adaptation and Pollination Biology

**DOI:** 10.1002/advs.202417054

**Published:** 2025-03-26

**Authors:** Rangjun Shi, Pan Duan, Mengmeng Zhu, Rong Zhang, Zihua Zhao, Xin Nie, Hanhou He, Li Hou, Xianhui Wang

**Affiliations:** ^1^ State Key Laboratory of Integrated Management of Pest Insects and Rodents Institute of Zoology Chinese Academy of Sciences Beijing 100080 China; ^2^ CAS Center for Excellence in Biotic Interactions University of Chinese Academy of Sciences Beijing 100049 China; ^3^ Institute of Plant Protection Ningxia Academy of Agricultural and Forestry Sciences Yinchuan 750002 China; ^4^ Department of Plant Biosecurity & MARA Key Laboratory of Surveillance and Management for Plant Quarantine Pests, College of Plant Protection China Agricultural University Beijing 100193 China

**Keywords:** diapause, evolution, genome, insect, *toll* receptors

## Abstract

The leafcutter bee *Megachile rotundata* is the world's most intensively managed solitary bee, owing to its easy manipulation and high pollination efficacy. Here, a high‐quality chromosome‐level *M. rotundata* genome, covering 280.68 Mb is presented. A total of 10 701 genes are predicted, of which 93.06% are functionally annotated. Based on the new genome assembly, transposable elements, noncoding RNAs, as well as gene families associated with pollination biology and ecological adaptation are systematically characterized. Comparative genomic analysis shows a notable expansion of *Toll* gene family but the contraction of detoxification gene in *M. rotundata* genome. Surprisingly, these expanded *Toll‐1* genes and their downstream genes display abundant mRNA levels in diapausing prepupae. Additionally, diapausing prepupae show significantly upregulated expression of antimicrobial peptide genes and a higher survival rate after *Escherichia coli* exposure compared to nondiapausing prepupae, indicating an enhanced immune response during *M. rotundata* diapause. The *M. rotundata* genome provides an important foundation for understanding its ecological adaptation and optimizing its exceptional pollination efficiency in the future.

## Graphical abstract

The genome of *Megachile rotundata*, an extensively managed solitary pollination bee species, has been sequenced, covering 280.68 Mb and predicting 10 701 genes. The study reveals significant expansions of the *Toll* gene family and their abundant expression in diapause prepupae, highlighting enhanced immune responses during diapause. This genome serves as a valuable resource for understanding ecological adaptation and optimizing pollination efficiency.

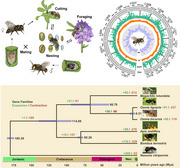

## 1 Introduction

The leafcutter bee *Megachile rotundata* F. (Hymenoptera: Megachilidae) is one of the most economically important managed solitary bee pollinators worldwide, primarily used for commercial pollination of alfalfa (*Medicago sativa* L.) (Fabaceae).^[^ ^1^, ^2^ ^]^ Females of *M. rotundata* can efficiently trip up to 80% of flowers they visit and significantly increase alfalfa seed yields.^[^ ^1^ ^]^ As a representative species of solitary bees, *M. rotundata* displays notable differences in neurophysiology, behavior, and ecological adaptability compared to honey bees and bumblebees, which have varying degrees of eusociality. This makes *M. rotundata* a special focus of studies exploring the evolutionary origins of bee sociality.^[^ ^3^ ^]^ Despite being classified as “solitary,” *M. rotundata* females prefer to nest in aggregation, establishing a critical biological foundation for their intensive management.^[^ ^2^ ^]^ Their high pollination efficiency and ease of management greatly enhance their successful domestication, making them key contributors to alfalfa seed production in North America and central Canada.^[^ ^1^, ^2^, ^4^ ^]^

**Figure 1:**
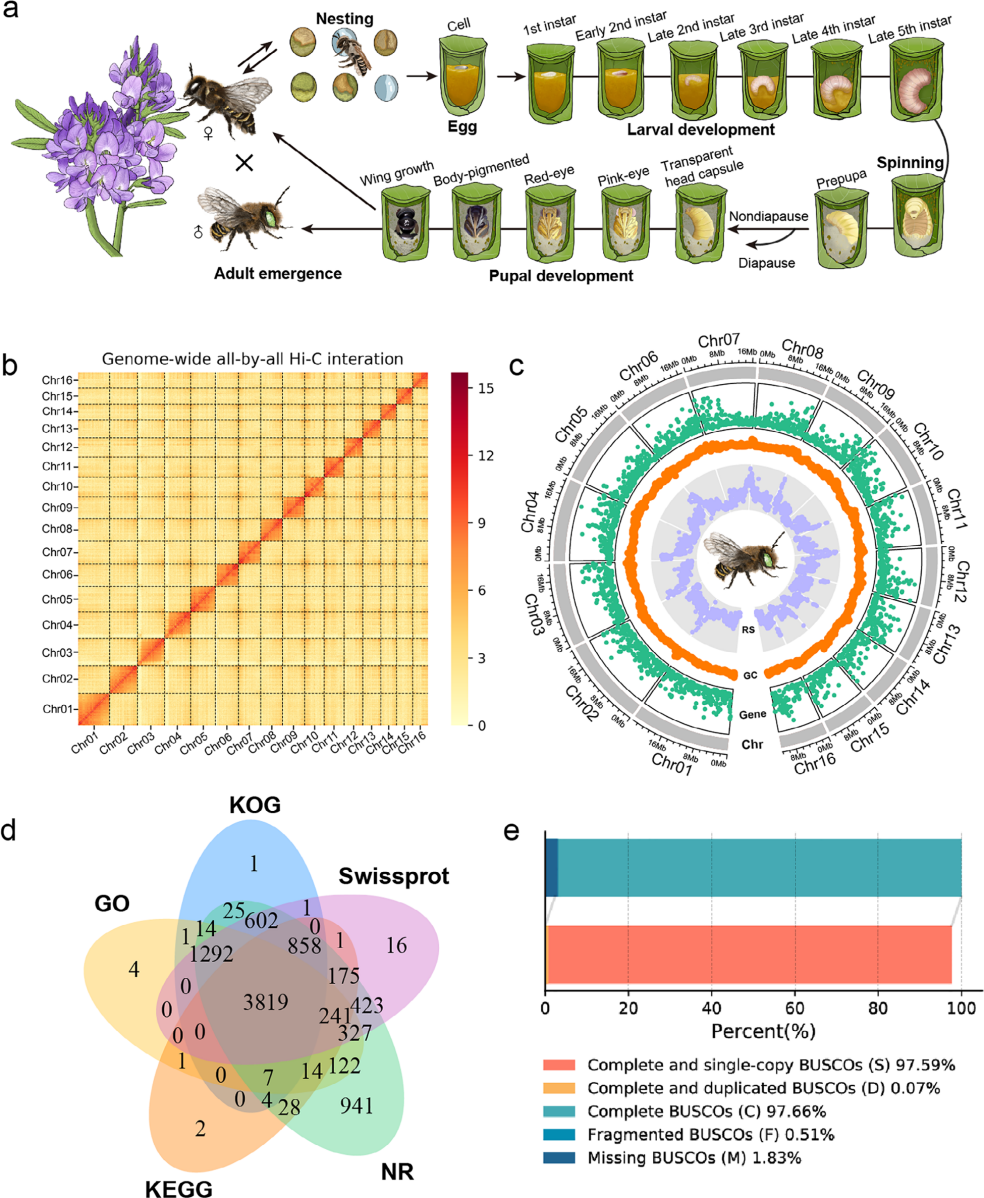
Chromosome‐level genome sequencing, assembly, and annotation. a) *M. rotundata* life cycle. Mated adult females cut leaves to construct cell cups and forage for pollen and nectar to create provision. The egg laid on the provision completes embryogenesis within several days and goes through five larval instars before entering the pupal stage. Diapause occurs at prepupal stage under maternal control, which may be influenced by various and interacting environmental factors. b) The heatmap of *M. rotundata* genomic Hi‐C interaction signals. c) Circos plot of genomic features for 16 chromosomes, with Chr (chromosome) from the inside out Gene (gene density), GC (GC content), and RS (repeat sequence). The bin size is 100 kb. d) Venn diagram illustrated the result of the number of genes annotated across different databases for *M. rotundata* OGS. e) Statistical results of BUSCO evaluation for the OGS.

*M. rotundata* is a cavity‐nesting pollinator exhibiting unique ecological adaptability. Females that emerge in the summer mate and construct linear nests made of individual cells, formed by cutting leaves and provisioning them with pollen and nectar.^[^ ^2^, ^5^ ^]^ A female bee lays an egg on the provision in each cell, which is then covered with additional leaf pieces as a cap.^[^ ^6^ ^]^ Each cell provisioning requires many foraging trips and returns to the nest of females, depending on precise olfactory and visual sensations and integration for nest positioning.^[^ ^2^, ^7^, ^8^ ^]^ Eggs complete embryogenesis within several days and develop into larvae in their brood cells until the fifth instar.^[^ ^9^ ^]^ *M. rotundata* prepupae experience facultative diapause, either entering diapause or averting it to emerge as adults; the following generation then enters diapause (**Figure** **1**a).^[^ ^7^ ^]^ The bivoltine nature of *M. rotundata* causes a significant deficit in leafcutting bee stock for its application in the next year. Diapause in *M. rotundata* may be influenced by various interactive environmental factors (e.g., day length, temperature, food provision), under maternal control.^[^ ^7^, ^10^, ^11^, ^12^, ^13^ ^]^ It has been shown that *M. rotundata* larvae are more susceptible to diseases, such as chalkbrood caused by the fungus *Ascosphaera aggregata*, leading to ≈60% mortality in developing bee brood.^[^ ^14^ ^]^ Consequently, diapausing prepupae in long‐term dormancy may face a high risk of infection. Clearly, understanding the molecular mechanisms behind *M. rotundata*’s ecological adaptation, such as nesting strategies, diapause control, and disease resistance, is vital for effective artificial breeding and advanced management practices.

A large number of studies have demonstrated the pollination performance of *M. rotundata*, along with the physiological and behavioral adaptations triggered by various environmental factors such as photoperiods, temperature, resource availability, and hypoxic conditions.^[^ ^7^, ^8^, ^15^ ^]^ However, to date, only a few studies have attempted to analyze gene expression patterns related to its ecological adaptation, with special attention to the diapause event. It has been proposed that diapause development involves a cascade of changes in thermal responses in *M. rotundata*. Using Northern blot, the mRNA levels of *HSC70* were shown to decrease, while the expression levels of *HSP70* and *HSP90* significantly increased in diapausing *M. rotundata* prepupae transferred from 4 to 25 °C.^[^ ^16^ ^]^ In addition, exposure to fluctuating temperatures leads to increased expression of genes involved in metabolic regulation, oxidative stress, immune response, and neurogenesis, which may contribute to increased longevity during chilling in *M. rotundata*.^[^ ^17^ ^]^ Furthermore, the expression levels of cyclin family and insulin pathway genes were altered by overwinter temperature and the timing of diapause initiation.^[^ ^18^, ^19^ ^]^ A recent transcriptome analysis shows that females can adjust mRNA and miRNA levels in maturing oocytes in response to seasonal changes, but no significant differences in maternal RNAs were observed in eggs laid within 24 h.^[^ ^20^ ^]^ In fact, little information is available on the molecular mechanisms underlying adaptive biology and pollination in *M. rotundata*, due to the absence of a complete genome, genetic tools, and sustained indoor breeding.

Here, we *de novo* assembled the first high‐quality, chromosome‐level *M. rotundata* genome using the Oxford Nanopore, PacBio HiFi, and Illumina platforms, assisted by the high‐throughput chromosome conformation capture (Hi‐C) technique. We annotated key gene families essential for pollination, nesting, and ecological adaptation, and analyzed their expression levels throughout the entire developmental process by performing RNA‐seq. Furthermore, we conducted comparative genomics analysis to reveal the genetic basis underlying its ecological adaptation related to pollination and disease resistance. Finally, we compared the expression response of Toll pathway genes between diapause and nondiapause prepupae upon bacterial challenge using quantitative reverse transcriptase‐polymerase chain reaction (qRT‐PCR).

## 2 Results

### 2.1 Chromosome‑Scale Genome Assembly and Annotation

Using 47.91 Gb of Illumina and PacBio Sequel2 data, the genome size of *M. rotundata* was estimated to have exceed 300 Mb through *k‐mer* analysis, and the heterozygosity was ≈0.50% (Figure S1a,b, Supporting Information). The preliminary assembly was constructed using 65.36 Gb of Nanopore ultralong reads (average length 39.12 kb), followed by multiple rounds of polishing with 29.17 Gb of PacBio HiFi reads and 18.74 Gb of Illumina data. The final polished genome demonstrated excellent accuracy (Single Nucleotide Accuracy of 99.99%) and completeness (99.27% and 97.98% in Benchmarking Universal Single‐Copy Orthologs (BUSCO) and CEGMA evaluations, respectively) (Figure S1c,d and Tables S1–S3, Supporting Information). Using 63.25 Gb of Hi‐C data, we obtained a chromosome‐level assembly and clustered scaffolds into 16 chromosomes based on karyotype analysis (2*n* = 32) in *M. rotundata* (Figure 1b,c).^[^ ^21^ ^]^ The Hi‐C interaction heatmap confirmed strong adjacent sequence interactions, resulting in a clear assembly (Figure 1b). The final chromosome‐level genome size was 280.68 Mb, containing 79 scaffolds with a 16.80 Mb N50 and 81 contigs with a 15.97 Mb N50 (**Table** **1** and Table S4, Supporting Information).

**Table 1:** Statistics of genome assembly and annotation results.

Features	*M. rotundata*
Assembly size [Mb]	280.68
Number of scaffolds/contigs	79/81
Longest scaffold/contig [Mb]	23.72/23.72
N50 scaffold/contig length [Mb]	16.80/15.97
Number of assembled chromosomes	16
Number of gaps	8
G + C [%]	36.69
Repeats [%]	32.82
Protein‐coding genes	10 701

We annotated repetitive elements in the *M. rotundata* genome, identifying 92.13 Mb of repeats, which account for 32.82% of the genome (Table 1 and Table S5, Supporting Information). This included 12 383 short tandem repeats (0.05%), 47 668 tandem repeats (3.44%), and 251 086 transposable elements (23.75%). Additionally, noncoding RNAs (ncRNAs) were predicted, resulting in a catalog of 163 rRNAs, 117 small RNAs, 110 regulatory RNAs, and 206 tRNAs (Table S6, Supporting Information).

Combining different *de novo* prediction methods, we generated the final Official Gene Set (OGS), which predicted 10 701 protein‐coding genes with an average gene length of 13.16 kb (Figure S2, Supporting Information). A total of 9958 (93.06%) genes were successfully annotated in five different databases, including 9916 (92.66%) in NR, 6204 (57.98%) in GO, 6985 (65.29%) in KOG, 6238 (58.29%) in KEGG, and 8326 (77.81%) in Swiss‐Prot (Figure 1d and Figures S3–S6, Supporting Information). BUSCO assessment indicated that ≈97.66% of complete gene elements were present in the *M. rotundata* OGS (Figure 1e). Additionally, 10 568 (98.76%) genes had mRNA support from transcriptome data across different developmental stages.

### 2.2 The Annotation of Gene Families Involved in Pollination Biology and Ecological Adaptation

Leafcutter bees spend most of their adult lives foraging and nesting. A complex genetic framework, involving chemical communication, visual processing, neurobehavioral regulation, and the ability to tolerate toxins or pathogens, is essential for their successful environmental adaptation.^[^ ^2^ ^]^ Therefore, we annotated and curated genes associated with vision, chemoreception, neuromodulation, and detoxification.

#### 2.2.1 Vision‐Related Genes

**Figure 2:**
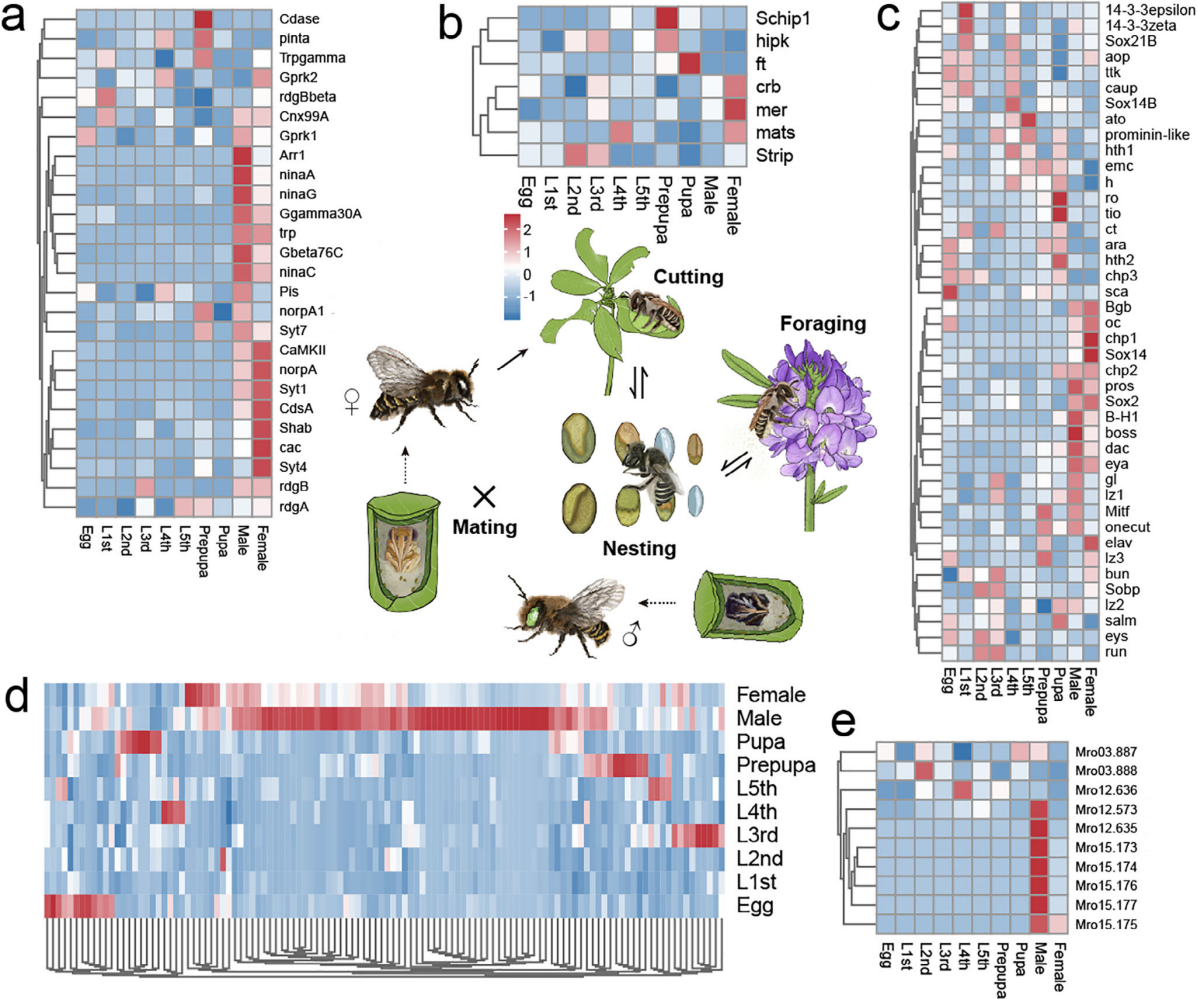
Expression patterns of gene families involved in vision and chemoreception in *M. rotundata*. The expression patterns of vision‐related genes, including a) phototransduction‐related genes, b) eye‐development‐related genes, and c) eye‐differentiation‐related genes. A series of genes related to vision were abundantly expressed at adult individuals. Developmental expression levels of d) odorant receptor genes (*ORs*), and e) odorant‐binding protein coding genes (*OBPs*). Seven *OBP* genes showed particularly high expression levels in male adults, while most *ORs* were also highly expressed in male adults.

Visual information has been shown to be of primary importance for nest location and foraging in various bees.^[^ ^22^, ^23^, ^24^ ^]^ Using the visual genes from fruit flies as seed sequences,^[^ ^25^, ^26^ ^]^ we annotated 75 vision‐related genes, which can be grouped into three main categories: eye development (42 genes), eye differentiation (7 genes), and phototransduction‐related genes (26 genes). Notably, most of the phototransduction genes were highly expressed during adulthood, with 8 male‐biased and 7 female‐biased genes (*Shab*, *cac*, *norpA*, *Syt1*, *Syt4*, *CdsA*, and *CaMKII*) (**Figure** **2**a–c). However, genes related to eye development and differentiation showed varied expression patterns throughout the entire developmental process. These results support the potential significance of phototransduction genes for adult‐specific activities, including tasks like foraging for food and locating nesting sites.

#### 2.2.2 Chemoreception‐Related Genes

The chemoreception system is vital for food finding, mate searching, and close‐range nest identification in *M. rotundata*.^[^ ^2^ ^]^ Different environmental cues are mainly recognized by three types of chemosensory receptor gene families: olfactory receptors (ORs), gustatory receptors (GRs), and ionotropic glutamate receptors (IRs). Odorant‐binding proteins (OBPs) bind and transfer odors to ORs.^[^ ^27^ ^]^ A total of 119 *ORs*, 8 *GRs*, 19 *IRs*, and 10 *OBPs* were curated in the *M. rotundata* OGS. Phylogenetic analysis of OR receptors from *M. rotundata* and the social bumblebee *Bombus terrestris* revealed six major evolutionary clades. Notably, Clade‐I, Clade‐II, and Clade‐VI exhibited significant contraction in *M. rotundata* (Figure S7a, Supporting Information). This contraction was the primary factor contributing to the difference in the number of *OR* gene families between the solitary *M. rotundata* and the social insect *B. terrestris*. Furthermore, the leafcutter bee odorant receptor coreceptor (Orco) was closely related to *B. terrestris* Orco and diverged from other *M. rotundata* olfactory receptors (Figure S7a, Supporting Information). We also compared copy numbers of four types of chemosensory receptor genes among six selected *Hymenoptera* species. The results showed that three solitary bee species had significantly fewer *OR* and *OBP* genes compared to social bees (Figure S7b, Supporting Information). Among them, 59 *OR* and 7 *OBP* genes were highly expressed in adult males (Figure 2d,e and Figure S7, Supporting Information), implying that high olfactory sensitivity might contribute to mate searching in males. By contrast, *IR* and *GR* genes showed varying expression patterns throughout the developmental process in *M. rotundata* (Figure S8a,b, Supporting Information).

#### 2.2.3 Neuromodulation‐Related Genes

The G protein‐coupled receptor (GPCR) superfamily is widely involved in the modulation of neural and behavioral plasticity.^[^ ^28^, ^29^ ^]^ We annotated 93 GPCR encoding genes in the *M. rotundata* OGS, which predominantly fell into three major families (Table S7, Supporting Information). GPCR Family‐1 (rhodopsin‐like family, PF00001) included 69 receptor genes, consisting of 6 opsins, 21 biogenic amine receptors, and 42 neuropeptide and protein hormone receptors. Family‐2 (secretin receptor family, PF00002) mainly consisted of secretin‐like receptors, such as diuretic hormone receptors and parathyroid hormone receptors. Finally, Family‐3 (metabotropic glutamate/pheromone family, PF00003) primarily contained seven‐transmembrane metabotropic glutamate receptors, γ‐aminobutyric acid receptors, and bride‐of‐severity receptors (an orphan G protein‐coupled receptor BOSS, which is a glucose‐responding membrane receptor).

Based on transcriptome data, we found that four opsin‐encoding genes (*Mro03.575*, *Mro10.174*, *Mro12.226*, and *Mro12.227*) were extremely highly expressed in adult males, and a large number of biogenic amine receptors displayed abundant expression levels in adult leafcutter bees (Figure S9, Supporting Information). By contrast, the genes encoding neuropeptide and protein hormone receptors showed diverse transcription patterns throughout the entire developmental process (Figure S9, Supporting Information). For the secretin‐receptor family, five members (*Mro01.61*, *Mro04.631*, *Mro07.370*, *Mro09.296*, and *Mro15.359*) showed relatively higher expression in prepupae, whereas some members (e.g., *Mro01.87*, *Mro12.91*, *Mro09.122*) were highly expressed in pupae. Most genes from the metabotropic glutamate/pheromone family showed significantly higher expression in both sexes of adults. The molecular characterization and expression pattern analysis of GPCRs lay the foundation for further exploration of the molecular basis underlying complex behaviors in *M. rotundata*.

#### 2.2.4 Detoxification Genes

The detoxification system consists of various detoxifying enzymes, such as cytochrome P450 (P450), glutathione‐S‐transferase (GST), and carboxyl/cholinesterase (CCE), and is crucial for insects to overcome toxins commonly found in the environment. We annotated 43 P450, 18 CCE, and 13 GST encoding genes in the *M. rotundata* OGS. *M. rotundata* had fewer P450 genes compared to five other *Hymenoptera* insect species, consistent with the loss of CYP9Q subfamily members in this species, as reported previously (Figure S10a, Supporting Information).^[^ ^30^ ^]^ Meanwhile, the number of *CCEs*, but not *GSTs*, in three *Megachilidae* species was lower than in *Apis mellifera* and *B. terrestris*. Transcriptome data showed that *CYP* genes mainly displayed three types of expression patterns: 15 of them showed larvae‐abundant levels, 16 genes were highly expressed at late larval or prepupal stages, and 15 genes exhibited high expression at the adult stage (Figure S10b, Supporting Information). Additionally, 4 *CCE* and 5 *GST* genes showed adult‐biased expression (Figure S10c,d, Supporting Information). The stage‐specific expression patterns of detoxifying enzyme genes suggest their distinct roles in processing varied food resources at different stages of the life cycle. Overall, the contraction of *P450* and *CCE* genes reinforces the notion that *M. rotundata* is more vulnerable to toxins or xenobiotics, highlighting the critical need for enhanced environmentally friendly pest management during *M. rotundata* pollination.

### 2.3 Genome Evolution through Chromosome Fusion and Fission Events in the *Megachile* Lineage

**Figure 3:**
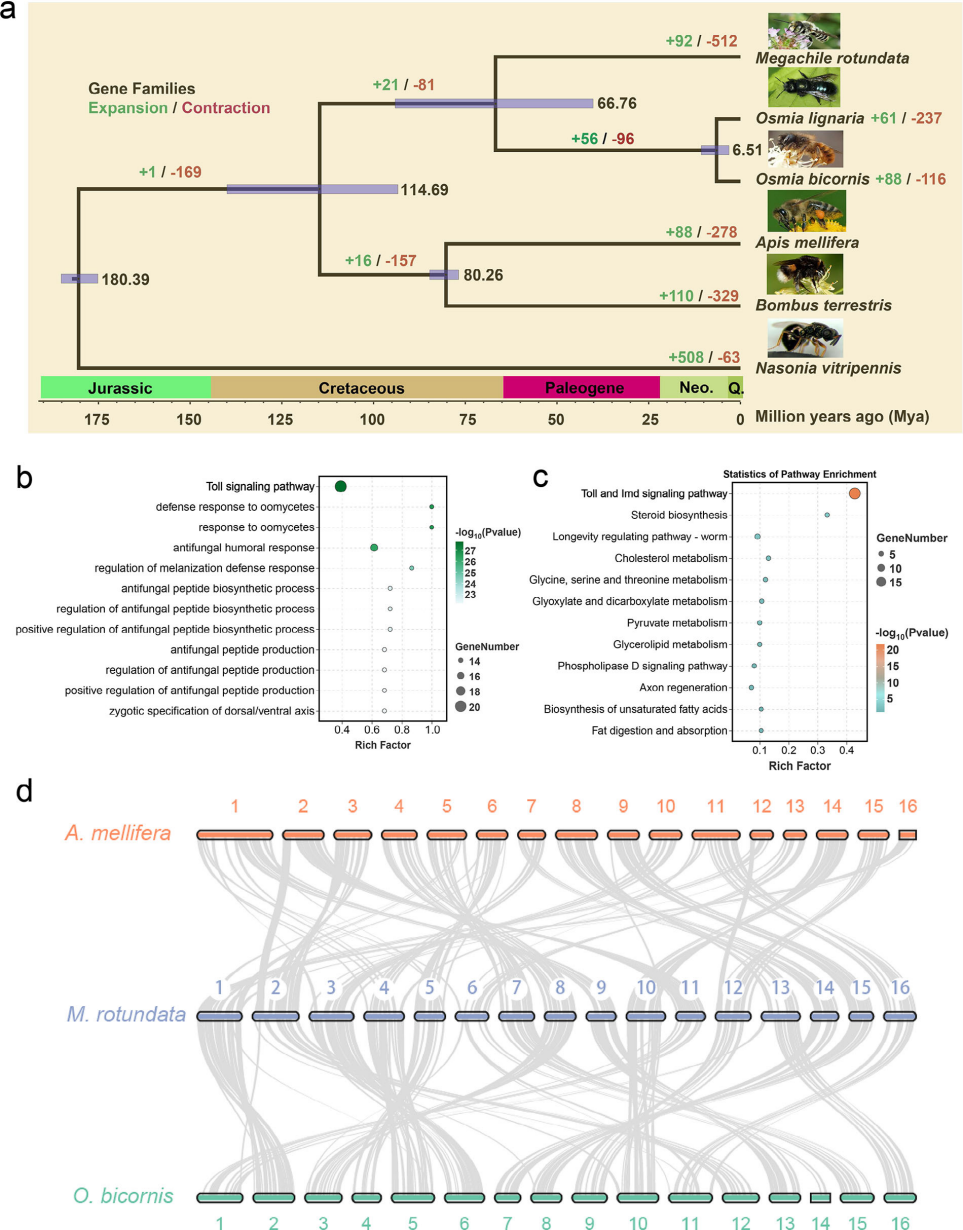
Gene family's evolution between genomes of *M. rotundata* and other hymenopteran species. a) A phylogenetic tree constructed based on single‐copy genes of *M. rotundata* and other hymenopteran species. The green and red represent the number of expanded and contracted orthogroups, respectively. The scale below represents the timeline of species divergence, while the black numbers on the internal nodes represent the divergence time estimated by MCMCTree. The blue box denotes the 95% highest posterior density (HPD). b) GO enrichment of significant expanded gene families in *M. rotundata* (*p* < 0.05, top 12). c) KEGG pathway enrichment of significant expanded gene families in *M. rotundata* (*p* < 0.05, top 12). d) Genomic synteny analysis of honeybee, red mason bee, and the leafcutter bee.

To understand the evolutionary history of the solitary bee *M. rotundata*, we first identified 6636 single‐copy orthologues in *M. rotundata* and five other representative *Hymenoptera* species with sequenced genomes. We then constructed a phylogenomic tree and estimated divergence times across the six selected species (**Figure** **3**a). *M. rotundata* diverged from its most closely related *Osmia* species, *Osmia bicornis*, as well as *Osmia lignaria*, around 66.76 Mya (95% highest posterior density (HPD): 40.1573–94.1018). The megachilid bees separated from other Apidae, such as *A. mellifera* and *B. terrestris*, ≈114.69 Mya (95% HPD: 93.3557–139.941) during the Cretaceous period. Our age estimates for megachilid bees are younger than those reported in previous studies.^[^ ^3^ ^]^ The Most Recent Common Ancestor of parasitoid wasps, honeybees, and megachilid bees (*M. rotundata*, *O. bicornis*, and *O. lignaria*) appeared around 180.39 Mya (95% HPD: 175.141–185.127) during the Jurassic period.

To track the chromosome evolution of *M. rotundata*, we compared it with the most closely related *O. bicornis* and the eusocial bee *A. mellifera*, both with chromosome‐level genomes (Figure 3d). Syntenic block analysis showed that *M. rotundata* shared most linear syntenic blocks with *O. bicornis*, *A. mellifera*, and *B. terrestris*, followed by *Nasonia vitripennis*, consistent with the phylogenetic relationships among these species (Figure S11a–d, Supporting Information). *M. rotundata* shared the same chromosome number with *A. mellifera* and *O. bicornis*. However, chromosome fusion and fission events occurred during evolution, especially on longer chromosomes (Figure S11e, Supporting Information). The *M. rotundata* chromosomes Chr01, Chr02, Chr03, Chr06, and Chr10 likely evolved from *A. mellifera* chromosome fusion events but underwent fission events subsequently, producing *O. bicornis* chromosomes. Moreover, the *M. rotundata* Chr04, Chr05, Chr12, and Chr14 chromosomes evolved from *A. mellifera* chromosome fusion events and remained relatively stable in synteny with *O. bicornis*. The *M. rotundata* Chr08 chromosome maintained a high degree of synteny with *A. mellifera* Chr04 and *O. bicornis* Chr03. Additionally, the *M. rotundata* Chr13 evolved from *A. mellifera* Chr08 and then split into Chr01 and Chr16 in *O. bicornis*. By contrast, the *M. rotundata* Chr07, Chr09, Chr11, Chr15, and Chr16 chromosomes appeared to be the products of chromosome fission events. Taken together, the chromosomes undergo structural rearrangements, but no changes in chromosome number occur from *A. mellifera* to *O. bicornis*.

### 2.4 Comparative Genomic and Phylogenomic Analyses Revealed the Expansion of *MroToll* Gene Family in *M. rotundata*

The solitary *M. rotundata* exhibits distinct physiological and behavioral traits compared to social bees. To further elucidate the genetic basis underlying the unique biology of *M. rotundata*, we identified contraction and expansion events across six different *Hymenoptera* species with varied degrees of sociality, including *M. rotundata*, *O. lignaria*, *O. bicornis*, *A. mellifera*, *B. terrestris*, and *N. vitripennis*. A total of 8507 orthogroups showed fluctuations in numbers, with 663 families exhibiting significant expansion or contraction (*p*‐value < 0.05). In *M. rotundata*, 512 families underwent contraction, while 92 families experienced expansion (Figure 3a). Of these, 55 rapidly expanding *M. rotundata* families (*p*‐value < 0.05) were subjected to functional enrichment. GO enrichment analysis revealed that the most significantly expanded *M. rotundata* gene families are associated with immunity and resistance to pathogenic microorganisms. These include the Toll signaling pathway, defense response to oomycetes, response to oomycetes, and antifungal humoral response (Figure 3b). KEGG enrichment further confirmed the significant expansion of genes involved in Toll and Imd signaling pathways (Figure 3c).

**Figure 4:**
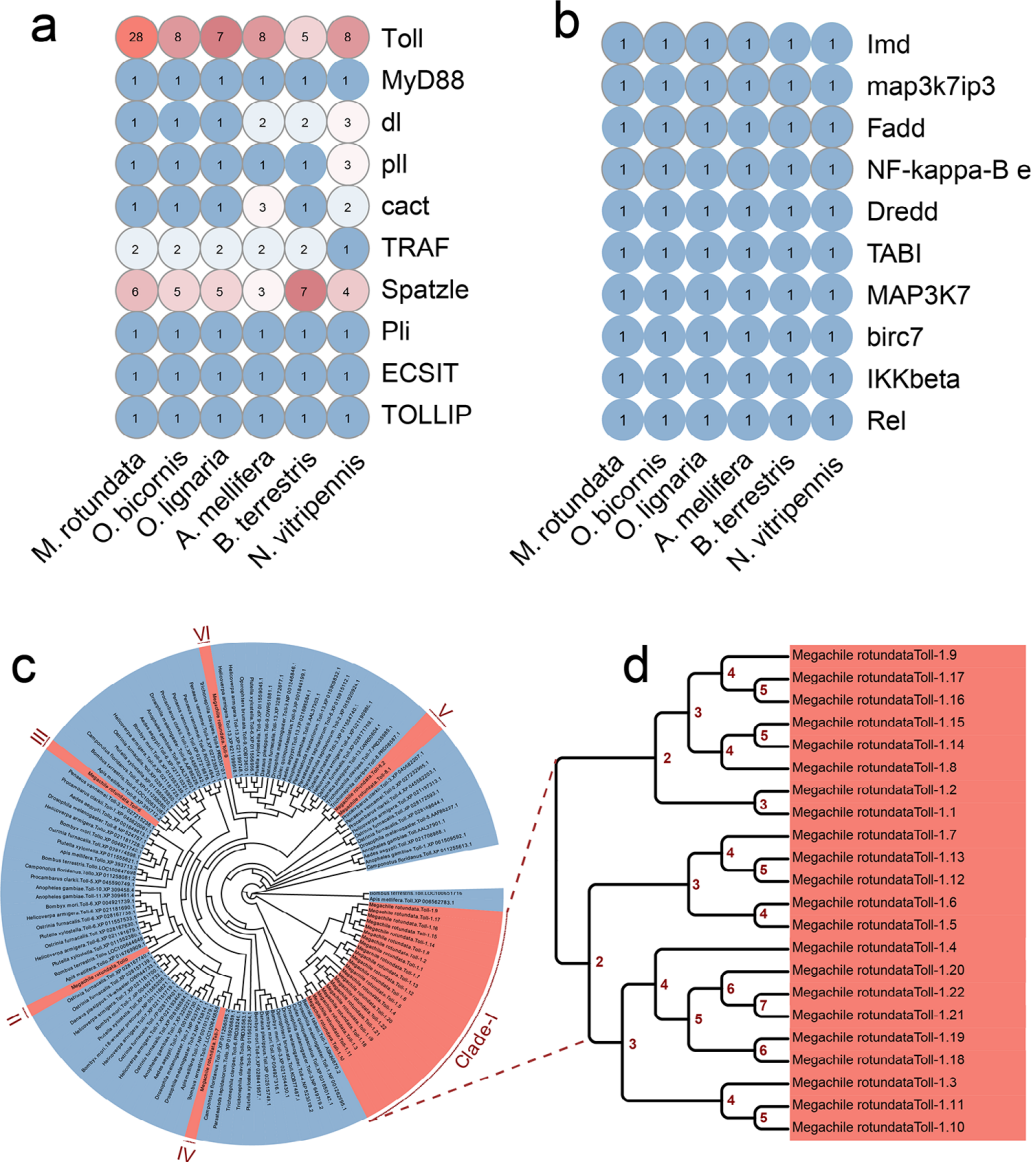
Evolution patterns of immune‐related genes in *M. rotundata*. a) The number of Toll signal pathway genes and b) the number of Imd signal pathway genes in *M. rotundata* and other arthropod species. NF‐kappa‐B e represents NF‐kappa‐B essential modulator. c) Phylogenetic relationship of Toll receptors in *M. rotundata* and other arthropod species. Toll receptors of the leafcutter bee can be divided into 6 major clades. Red background represents the different clades of MroTolls. The MroToll‐1 clade showed significant expansion (Clade‐I). d) Standardized renaming of *MroToll* genes in *M. rotundata*. The largest clade contained 22 *MroTolls*, which were clustered on the same branch with *D. melanogaster Toll‐1*, are named *MroToll‐1.1–MroToll‐1.22* according to their numbering in the evolutionary tree nodes. The *Mro03.638*, *Mro03.687, Mro02.218*, and *Mro02.618* were renamed as *MroTollo*, *MroToll‐6*, *MroToll‐7*, and *MroToll‐9*, respectively, according to their phylogenetic relationship with homologs in *B. terrestris*, *A. mellifera*, and *D. melanogaster*. Moreover, two *MroTolls* (*Mro04.412* and *Mro06.106*) belonged to the same clade as *Toll‐7* and *Toll‐8* in the spider *T. clavipes*. Since *Toll‐7* already had a dedicated evolutionary branch, these two *MroTolls* were collectively named the *MroToll‐8* group.

To validate whether genes related to immunity experience expansion in *M. rotundata*, we further annotated all genes in the Toll and Imd signaling pathways. The *M. rotundata* genome contained nearly all key molecular components of the Toll and Imd pathways (**Figure** **4**a,b). There are 28 Toll receptor genes in the *M. rotundata* OGS. Additionally, a total of 15 genes in the Toll pathway and 10 genes in the Imd signaling pathway were characterized. However, only three AMP‐encoding genes were annotated in the *M. rotundata* OGS, including two *MroDefensins* (*MroDefensin 1* and *MroDefensin 2*) and one *MroApidaecin*. Some AMPs commonly found in fruit flies and honeybees are absent in the leafcutter bee genome.

Insects have multiple Toll receptors that display distinct physiological functions, such as morphogenesis, locomotion behavior, neuronal survival, and olfactory circuits.^[^ ^31^, ^32^, ^33^, ^34^ ^]^ To classify the phylogenetic relationships of Toll members in *M. rotundata*, we constructed a phylogenetic tree using Toll receptors from *M. rotundata* and 18 other arthropod species. Based on the phylogenetic tree, the MroToll receptors were classified and nominated as MroToll‐1 (Clade I), MroTollo (Clade II), MroToll‐6 (Clade III), MroToll‐7 (Clade IV), MroToll‐8 (Clade V), and MroToll‐9 (Clade VI) (Figure 4c). Notably, there were 22 MroToll members, which clustered with *Drosophila melanogaster* Toll‐1, and were thus renamed the Toll‐1 group (MroToll‐1.1 to MroToll‐1.22), indicated that MroToll‐1 had undergone significant gene expansion events (Figure 4c,d).

### 2.5 Diapausing Prepupae Have Higher *MroToll‐1* Expression and Stronger Immune Response upon Bacterial Challenging

**Figure 5:**
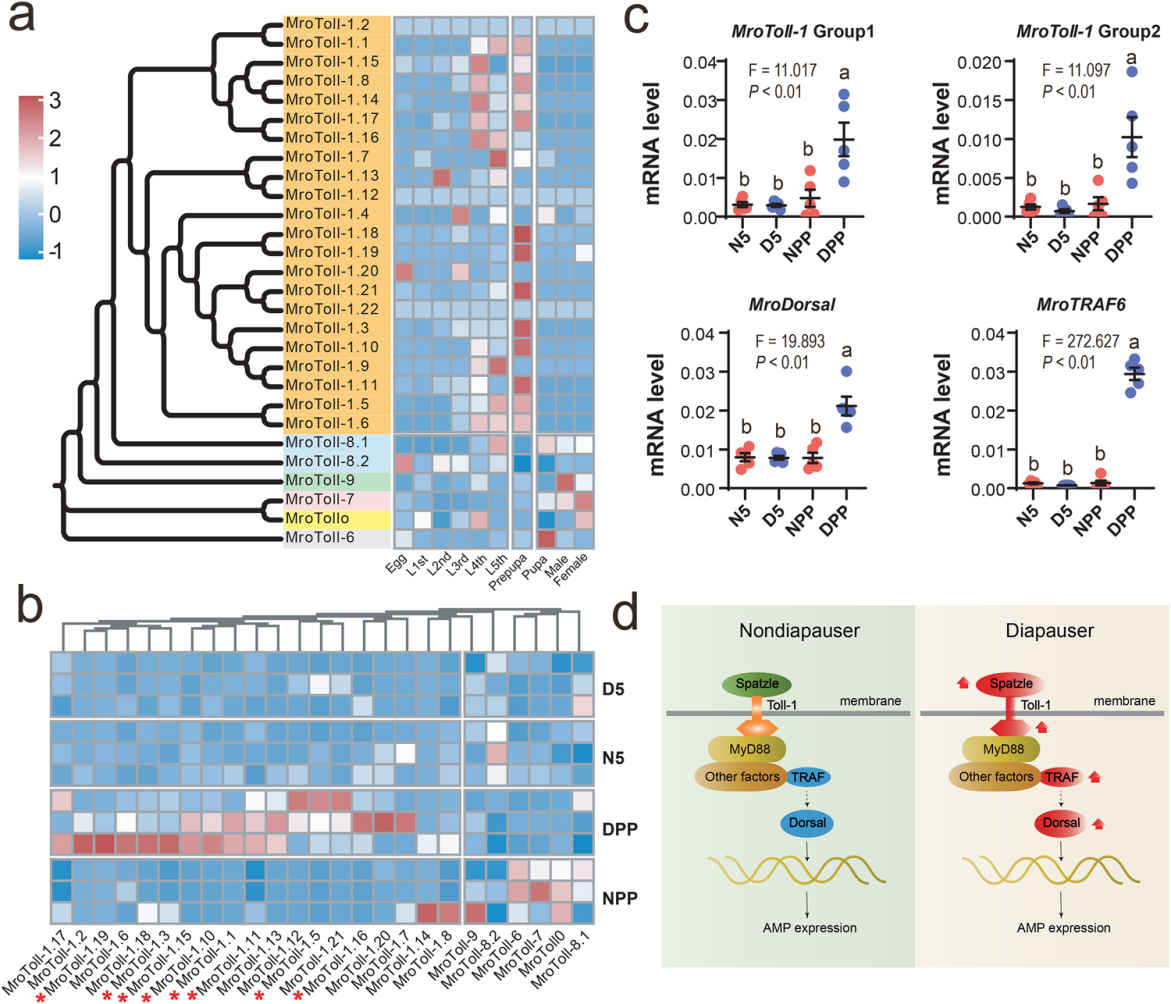
Expression patterns of Toll signal pathway genes in *M. rotundata*. a) The expression patterns of *MroToll* genes at different developmental stages in the leafcutter bee. *MroToll‐1* clade genes (orange) were mainly highly expressed during the later larval stage, especially during the prepupal stage. L1st – L5th represented the larval stages from 1st to 5th instar larvae. b) Expression patterns of *MroToll* genes in the 5th instar larvae and prepupae (PP) under diapause (D) and nondiapause (ND) states. *MroToll‐1* group genes were highly expressed in the diapause prepupal stage. The *MroToll* genes marked with red stars were significantly upregulated in diapausing prepupae compared to nondiapause individuals (FDR < 0.05, FC > 1.5). c) Comparative expression analysis of key Toll signal pathway genes between diapausing and nondiapausing *M. rotundata* bees. N5/D5: nondiapausing (N)/diapausing (D) 5th instar larvae. N/DPP: nondiapausing (N)/diapausing (D) prepupae (PP). Among them, *MroToll‐1* Group1 and Group2 represent the quantitative qPCR expression results of the two major *MroToll‐1* groups in diapausing, nondiapausing 5th instar larvae and prepupae. d) Schematic diagram showing the expression levels of genes involved in Toll signaling pathway between diapausing and nondiapausing *M. rotundata* prepupae.

To explore the potential role of the expanded *Toll* genes in *M. rotundata*, we performed transcriptome analysis of the entire developmental process in the leafcutter bee, including eggs, 1st to 5th instar larvae, prepupae, pupae, and newly emerged adult females and males (Figure 1a). The results showed that most of the *MroToll‐1* genes exhibited higher mRNA abundance during the final instar larval stage (5th instar) and the prepupal stage (**Figure** **5**a). Because the transition from the 5th instar to prepupae is a critical time window for diapause occurrence in *M. rotundata*,^[^ ^2^ ^]^ we further compared the expression profiles of *MroToll* genes in 5th instar larvae and prepupae between diapause and nondiapause states. Notably, eight of the 19 expressed *MroToll‐1* subfamily genes displayed significantly higher mRNA levels in diapausing prepupae compared to nondiapausing prepupae (Figure 5b).

Multiple sequence alignments of these eight *MroToll‐1* genes revealed high identity among *MroToll‐1.1*, *MroToll‐1.15*, and *MroToll‐1.16* (*MroToll‐1* group 1), while *MroToll‐1.5*, *MroToll‐1.10*, *MroToll‐1.11*, *MroToll‐1.13*, and *MroToll‐1.19* (*MroToll‐1* group 2) shared highly conserved sequences (Figure S12, Supporting Information). To validate the expression patterns, we performed qPCR using universal primers designed from the common sequences of each group of *MroToll‐1* genes. The qPCR results indicated that both *MroToll‐1* group 1 and group 2 genes were more abundantly expressed in diapausing prepupae than in 5th instar larvae and nondiapausing prepupae (Figure 5c).

Additionally, we examined the expression patterns of other key genes in the Toll pathway, including signal transduction molecules and downstream AMPs. We found that *MroSpaetzle*, *MroDorsal*, and *MroTRAF6* were highly expressed in diapausing prepupae, whereas the expression levels of the three AMP genes (*MroDefensin 1*, *MroDefensin 2*, and *MroApidaecin*) did not differ between diapausing and nondiapausing prepupae (Figure 5c,d and Figure S13, Supporting Information). Considering the conserved role of the Toll signaling pathway in innate immunity,^[^ ^35^, ^36^, ^37^, ^38^ ^]^ the high expression levels of *MroToll‐1* genes might indicate their potential role in pathogen defense during diapause.

**Figure 6:**
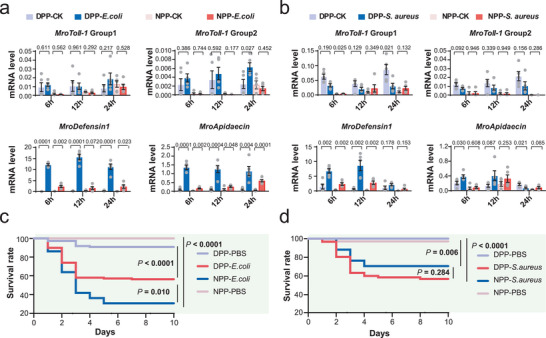
Expanded *MroToll* genes may contribute to pathogen defensing in diapausing prepupae of *M. rotundata*. a) The mRNA levels of *MroToll‐1* and antimicrobial peptide (AMP) encoding genes in diapausing and nondiapausing prepupae upon infection with *E. coli*. b) The mRNA levels of *MroToll‐1* and AMP encoding genes in diapausing and nondiapausing prepupae upon infection with Gram‐positive *S. aureus*. *p* values were labeled above each group of compared columns. Data were presented as mean ± standard error of the mean (SEM). Five biological repeats were contained in each treatment. c) Survival rate of diapausing and nondiapausing prepupae upon infection with *E. coli* bacteria. d) Survival rate of diapausing and nondiapausing prepupae upon infection with *S. aureus*. Survival rates were analyzed with the log‐rank (Mantel–Cox) test. At least 20 individuals were included in each treatment.

To test whether these *MroToll* genes were associated with immune response, we analyzed temporal changes in mRNA levels of two *MroToll‐1* group genes, *MroDorsal*, *MroTRAF6*, as well as three AMP‐encoding genes (*MroDefensin‐1*, *MroDefensin‐2*, and *MroApidaecin*) in diapausing or nondiapausing prepupae following infection with Gram‐negative (*Escherichia coli*) or Gram‐positive (*Staphylococcus aureus*) bacteria. Among the seven genes detected in the qPCR assay, *MroDefensin‐1* and *MroApidaecin* exhibited the most pronounced gene expression changes upon bacterial infection (**Figure** **6**a,b). Infection with *E. coli* resulted in a dramatic increase in *MroDefensin‐1* mRNA levels at all three time points, with a 143.2‐fold, 74.8‐fold, and 37.2‐fold increase in diapausing prepupae post 6, 12, and 24 h, respectively, and a 16.8‐fold, 5.2‐fold, and 15.7‐fold upregulation in nondiapausing prepupae at the same time points (Figure 6a). Infection with *S. aureus* also led to significantly increased expression of *MroDefensin‐1* in both diapausing and nondiapausing prepupae, with a 4.5‐fold, 15.6‐fold, and 1.9‐fold increase in diapausing prepupae after 6, 12, and 24 h, respectively, and a 9‐fold, 13.3‐fold, and 7.4‐fold increase in nondiapausing prepupae (Figure 6b). *MroApidaecin* also displayed clear induction after *E. coli* injection. Its mRNA level increased by 15.4‐fold, 12.9‐fold, and 13.0‐fold in diapausing prepupae post 6, 12, and 24 h *E. coli* injection, respectively, and by 5.6‐fold, 2.7‐fold, and 10.5‐fold in nondiapausing prepupae at the same time points (Figure 6a). By contrast, *S. aureus* infection only slightly stimulated *MroApidaecin* gene expression in diapausing prepupae at 6 and 24 h, and did not change expression levels in nondiapausing prepupae at any time point (Figure 6b). Additionally, *MroDorsal* mRNA levels were significantly elevated in diapausing prepupae following *E. coli* infection (3.1‐fold, 6.6‐fold, and 4.2‐fold increase at 6, 12, and 24 h, respectively), but no changes were observed in nondiapausing prepupae (Figure S14b, Supporting Information). Infection with *S. aureus* similarly increased *MroDorsal* mRNA levels in both diapausing and nondiapausing prepupae (1.8‐fold for diapause and 1.5‐fold for nondiapause) after 12 h (Figure S14a, Supporting Information). The *MroToll‐1* group genes and *MroTRAF6* gene showed no significant expression changes in response to either *E. coli* or *S. aureus* infection (Figures 6 and Figure S14, Supporting Information). Notably, the AMP‐encoding gene *MroDefensin‐2* exhibited decreased expression in diapausing prepupae upon *E. coli* injection, suggesting an alternative role in bacterial immune response. Overall, these data indicate that AMP genes exhibit stronger expression changes in diapausing prepupae compared to nondiapausing prepupae, particularly in response to *E. coli* infection, implying potential differences in immune competence between diapausing and nondiapausing prepupae.

We further determined the survival rate of diapausing and nondiapausing prepupae following infection with *E. coli* or *S. aureus*. After ten days of *E. coli* infection, both diapausing and nondiapausing prepupae had significantly lower survival rates compared to the phosphate‐buffered‐saline (PBS)‐injected controls (90.9% and 100% survival for diapausing and nondiapausing groups, respectively). However, the survival rate of diapausing prepupae (56.8%) affected by *E. coli* was much higher than that of nondiapause individuals (28.6%) (Figure 6c). After *S. aureus* infection, the survival rate of diapausing prepupae and nondiapausing prepupae dropped to 56.6% and 70.6%, respectively, while the PBS controls maintained high survival rates (100% for diapausing prepupae and 97% for nondiapausing prepupae, respectively) (Figure 6d). These findings suggest that diapausing prepupae may possess stronger immune defenses against pathogens.

## 3 Discussion

As the most intensively managed nonsocial bee, *M. rotundata* presents an excellent model for the commercialization of other solitary bees. Although its natural history and biological traits have been widely reported,^[^ ^2^ ^]^ the genetic mechanisms underlying its successful management and pollination characteristics are largely unknown. We employed a combination strategy to generate a 280.68 Mb *M. rotundata* chromosome‐level genome assembly. The high quality of the newly assembled genome was supported by its high accuracy, continuity, and completeness, and was reflected in a much longer contig N50 length (15.97 Mb) compared to the previously released *M. rotundata* scaffold genome (MROT_1.0, 272.7 Mb, contig N50 64.2 kb) (University of Maryland, 2011). So far, more and more bee genomes have been assembled using third‐generation sequencing technologies, with few species of the *Megachile* genus, which accounts for one‐third of all megachilids.^[^ ^39^ ^]^ Therefore, the chromosome‐level genome assembly provides fundamental genetic resources for understanding *M. rotundata* biology and bee chromosome evolution.

A major finding of the current study is that the leafcutter bee genome has an expanded Toll receptor gene family, with 28 Toll receptor genes, about 3 and 5 times the number in honeybees and bumblebees, respectively. Among the six subfamilies of the *MroToll* receptor genes, only the *Toll‐1* subfamily genes show significant expansion. The number and functions of Toll receptors in insects vary depending on species and receptor subtypes. In *Drosophila*, there are 9 Toll‐related genes showing diverse roles in regulating development, behavior, and neurocircuit assembly.^[^ ^40^, ^41^ ^]^ Of them, Toll‐1 is the most well‐investigated member due to its conserved importance in immune defense.^[^ ^35^, ^36^ ^]^ Interestingly, most of the *MroToll‐1* genes and key components in the Toll pathway (e.g., *Spaetzle*, *Dorsal*, and *TRAF*) exhibited much more abundant expression levels in diapausing prepupae. Unlike other bee species, *M. rotundata* experiences diapause in its larval stage, during which the larvae have more fragile epidermises that provide insufficient protection from pathogen infection. Therefore, a heightened immune function might compensate for the inadequacy of the epidermal barrier to protect *M. rotundata* from pathogen invasion.

We further revealed that diapausing prepupae display much stronger expression induction of antimicrobial peptides and better viability compared to nondiapausing prepupae after stimulation by *E. coli* but not *S. aureus*. The Toll pathway plays a conserved role in immune defense, especially against Gram‐positive bacteria and fungi, by acting as pattern‐recognition receptors and promoting antimicrobial peptide synthesis and secretion.^[^ ^36^, ^42^ ^]^ Therefore, diapausing prepupae may employ a more efficient resistance strategy in response to infection by distinct pathogenic microorganisms. Like other intensively managed bees, *M. rotundata* larvae are more susceptible to disease pathogens, such as chalkbrood disease induced by the fungus *Ascosphaera aggregate*.^[^ ^14^ ^]^ Sustained hibernation in the late larval stage will inevitably elevate the probability of being infected by fungi and other pathogens. As observed in social bees, *M. rotundata* has lost most of the common antimicrobial peptide genes and only maintains the classical Imd pathway effector defensins and a bee‐specific apidaecin peptide. It has been shown that apidaecins and defensins are activated by the Toll pathway, whereas abaecin and hymenoptaecin are activated by the Imd pathway in honeybees.^[^ ^43^ ^]^ Given the primary function of Toll receptors in pathogen recognition and immune signaling activation,^[^ ^35^, ^36^ ^]^ the higher level of *MroToll‐1* genes may facilitate the recognition and sensing of pathogens at a lower threshold (e.g., *Ascosphaera aggregate*), effectively activating antimicrobial molecules to defend against microorganisms in diapausing individuals of *M. rotundata*. In most insects, signal transduction of the Toll pathway depends on MyD88, a TIR domain‐containing molecule that activates AMP expression via TRAF and NF‐κB members (e.g., Dorsal).^[^ ^44^, ^45^ ^]^ In *M. rotundata*, *MroTRAF6* and *MroDorsal* display much higher expression levels in diapausing prepupae, indicating their potential involvement in enhanced AMP expression upon microorganism infection. Further studies, such as RNAi of *MroToll‐1* and downstream genes, are required to determine the distinct roles of Toll pathway members in pathogen defense in *M. rotundata*.

Comparative genomic studies have demonstrated that a significant contraction of detoxifying enzymes, in particular the P450 family genes, which are crucial for insects to degrade xenobiotic compounds and toxins ingested from food resources, as well as to mitigate the effects of insecticides exposure in the environment.^[^ ^46^ ^]^ This finding is further supported by a previous study revealing the absence of the specific CYP9Q P450 subfamily or the closely related genes, resulting in extremely low tolerance of *M. rotundata* to neonicotinoids.^[^ ^30^ ^]^ Although CYP9Q subfamily members are lost in all three selected solitary bee species, including *M. rotundata*, *O. lignaria*, and *O. bicornis*, the latter two *Osmia* species retain the CYP9Q‐related genes (CYP9BUs), which may confer the ability to detoxify pesticide thiacloprid.^[^ ^47^ ^]^ Given the broader decline in bee pollinator populations,^[^ ^48^, ^49^ ^]^ special care should be taken with pesticide applications to prevent unintended harm to the leafcutter bee populations. The P450 enzymes have been shown to play species‐distinct roles in the biosynthesis and modification of various pheromones in insects.^[^ ^50^, ^51^ ^]^ Therefore, one possible explanation for the loss of CYP9 subfamily gene members in three solitary bee species is their reduced reliance on pheromones‐dependent chemical communication among individuals. Additionally, *M. rotundata* and the two *Osmia* species possess fewer CCEs compared to social bee species. The CCE superfamily is another critical class of detoxifying enzymes, responsible for hydrolyzing carboxylic esters to alcohols and acids.^[^ ^52^ ^]^ Detoxification genes have been shown to undergo duplication or deletion events under dynamic selection pressures, such as host switching in herbivorous insects.^[^ ^53^ ^]^ Compared to honey bee and bumble bee, the leafcutter bee and mason bees exhibit a relatively narrow pollination spectrum and diet range, with the former primarily feeding on the pollen and nectar of leguminous plants (e.g., alfalfa), while the latter mainly consuming nectar and pollen from plants in the *Rosaceae* family.^[^ ^2^, ^54^ ^]^ Thus, the limited exposure to toxic plant secondary metabolites may be a key factor contributing to the contraction of detoxification enzyme gene in *M. rotundata* and two *Osmia* species.

Bee pollinators rely heavily on peripheral sensory cues for foraging, nesting, and mating.^[^ ^55^, ^56^ ^]^ However, the mechanisms underlying visual and chemical perception in *M. rotundata* remain poorly understood. We systematically annotated gene families associated with vision, olfaction, and gustation in *M. rotundata*, laying an important molecular foundation for exploring sensory‐mediated behaviors crucial for pollination. The leafcutter bees primarily use visual signals to select hosts and locate long‐distance nesting sites.^[^ ^2^, ^57^ ^]^ We found several eye‐development‐related genes, and most phototransduction genes showed high expression in adult females and males, indicating their role in visual‐cue‐mediated foraging behavior. For breeding, female *M. rotundata* must repeatedly travel to and from their nest, using visual landmarks for precise location.^[^ ^2^ ^]^ Notably, several phototransduction genes (e.g., *CaMKII*, *syt1*, and *syt4*) exhibited a female‐biased expression pattern in *M. rotundata*. The synaptotagmin (syt) and CaMKII proteins are known to play key roles in learning, memory, and synaptic plasticity.^[^ ^58^ ^]^ The abundance of these genes may suggest their involvement in vision‐based memory required for precise nest location.

Compared to social bees (e.g., *A. mellifera* and *B. terrestris*), the *M. rotundata* along with two other two solitary bee species (*O. lignaria* and *O. bicornis*) have much fewer *OR* and *OBP* genes, consistent with contraction of CYP subfamily genes that may be involve in pheromone synthesis, further suggesting reduced chemical communication among individuals in solitary bees. Notably, *M. rotundata* males express a large number of chemoreception genes (7 *OBPs* and 59 *ORs*). Generally, males emerge two days earlier than females and can mate multiple times, with a high sex ratio causing intense competition for successful copulation.^[^ ^59^ ^]^ The abundant expression of distinct olfactory receptors and related odor‐binding proteins in males may reflect an effective chemical communication system of males evolved for female location. Increasing evidence suggests that many *OR* and *OBP* genes are notably expressed in the male germ line during spermatogenesis, emphasizing their significance in spermatocyte maturation and sperm–oocyte chemiotaxis.^[^ ^60^, ^61^ ^]^ Given this, putative involvement of male‐abundantly expressed chemoreception genes in male reproduction cannot be excluded. Further studies are needed to elucidate the detailed roles of these genes in peripheral sensation or male reproduction, as well as their impact on pollination behavior in *M. rotundata*.

We have totally annotated 93 *M. rotundata * GPCRs which play critical roles in many aspects of physiology and behaviors.^[^ ^62^ ^]^ Several photoreceptor encoding genes (*opsins*) are abundantly expressed in adult males. It has been demonstrated that the bee color vision sensitivity differs between sexes, where females are sensitive to UV light for flower and nest site searching while males are sensitive to blue and green light for locating mates and territories.^[^ ^63^ ^]^ Therefore, the higher expression pattern of *opsins* in *M. rotundata* adult males indicates their potential participation in male‐specific vision perception. Biogenic amines act at important messenger in both central nervous system and peripheral organs, and modulate extensive behavioral repertoire through various amine‐specific receptors.^[^ ^64^, ^65^ ^]^ In *M. rotundata*, most of the identified amine receptor genes have high mRNA levels in adults exhibiting complex task‐related behaviors, including food collection, leaf cutting, mating, nesting, etc. Future studies should focus on the contribution of amine‐induced signaling cascades to the leafcutter bee behaviors. Moreover, gamma‐aminobutyric acid also has adult‐specific transcription pattern. Experimental tools (e.g., RNAi) should be employed to explore potential functions of these receptors in motor activities or olfactory learning as observed in honeybee.^[^ ^66^, ^67^ ^]^

In this study, we report the first high‐quality chromosome‐level genome assembly of the solitary bee *M. rotundata*. The expansion of Toll receptor genes and their extremely abundant expression in *M. rotundata* diapausing prepupae, imply its adaption to disease resistance during dormancy. The significant contraction of detoxification genes, especially for P450 and CCE genes, emphasizes its potential importance as environmental safety indicator. Overall, the chromosome‐level genome assembly constructed in this study significantly advances our understanding of *M. rotundata* genetics and facilitates future investigation into ecological adaption and efficient management of this solitary bee pollinator.

## 4 Experimental Section

### Sample Collection

Prepupae of *M. rotundata* collected from Pingluo, Ningxia Hui Autonomous Region, China, were maintained in 30 °C and 60% relative humidity. Genomic DNA was extracted from newly emerged haploid male adult using the Qiagen regent kit (Q13343) and sequenced using Nanopore, HiFi, and Hi‐C technology platforms. For transcriptome sequencing, whole bodies of eggs, larvae (from 1st to 5th larval stages), prepupae, pupae, and newly emerged adults (females and males) were separately collected for developmental gene expression analysis. The 5th instar larvae and prepupae under both diapause and nondiapause states were collected using a “reverse‐reasoning method” (descripted below) for diapause‐related transcriptome analysis. All samples were immediately frozen in liquid nitrogen, and stored at −80 °C. Two or more independent biological replicates were included for each transcriptome sample.

### Genome Sequencing and Assembly

PE150 DNA libraries with an insertion fragment size of ≈350 bp were constructed following the standard protocol, and sequenced on the illumina Novaseq platform by Nextomics Bioscience Co., Ltd. The sequencing depth was over 50 ×. After removing mitochondrial reads, *k‐mer* survey was performed to estimate genome size and heterozygosity rate.^[^ ^68^ ^]^ Nanopore ultralong pass reads were de novo assembled with NextDenovo v2.3.1 using the following steps: i) generating corrected sequence (CNS) using the NextCorrect module; ii) producing preliminary assembly of *M. rotundata* genome on the CNS using the NextGraph module; iii) genomic polishing using Pacbio HiFi reads and Illumina data. Genomic completeness of the *M. rotundata* genome (MROT_3.0) was then assessed using BUSCO and CEGMA pipelines. The accuracy was evaluated by using Burrows–Wheeler Aligner. Additionally, genomic contamination was assessed by mapping against the NT database using BLASTn. To assemble the chromosomes, the Hi‐C library was constructed following a well‐established protocol.^[^ ^69^ ^]^ Generated unique Hi‐C reads were mapped against the genome assembly using Bowtie2 v2.3.2. The valid Hi‐C data were then clustered, sorted, and oriented using LACHESIS software.^[^ ^70^ ^]^

### Genome Annotation

For protein‐coding gene prediction, three methods were used to annotate gene elements in *M. rotundata* genome by PASA, GeMoma, and AUGUSTUS. First, gene structure prediction was based on transcriptome data using GMAP, PASA, and GeneMarkS‐T.^[^ ^71^, ^72^, ^73^ ^]^ Homology species prediction was conducted using GeMoMa^[^ ^74^ ^]^ based on gene sets from *D. melanogaster* (GCF000001215.4), the Florida carpenter ant *Camponotus floridanus* (GCF003227725.1), *Tribolium castaneum* (GCF031307605.1), *N. vitripennis* (GCF009193385.2), *A. mellifera* (GCF003254395.2), the red mason bee *O. bicornis* (GCF004153925.1), *B. terrestris*, and the second‐generation *M. rotundata* genome MROT_1.0 (GCF000220905.1). Additionally, de novo prediction was performed by AUGUSTUS v3.3.1.^[^ ^75^ ^]^ Finally, the results were integrated using EvidenceModeler.^[^ ^72^ ^]^ The integrity of *M. rotundata* OGS was assessed by using BUSCO method. Expression levels of predicted genes were analyzed using transcriptome data. For function annotation, the predicted protein sequences were used to align against the NR, KEGG, KOG, and Swiss‐Prot databases using BLASTP. Additionally, GO annotations were obtained based on InterPro information using InterProScan. For repeat sequence annotation, GMATA and Tandem repeats finder were used to predict tandem repeats. The small transposon called MITE was predicted using MITE‐Hunter. The de novo search was performed using RepeatModeler, and the classification was generated using TEclass. For noncoding RNA prediction, three methods were performed: i) the Rfam database was used for prediction of rRNA, snRNA, and miRNA; ii) tRNAs were predicted using the software tRNAscan‐SE v2.0 (parameter: ‐ thread 4‐E‐I); iii) rRNAs were annotated using RNAmmer v1.2 (parameters: ‐ S euk‐m lsu, ssu, tsu gff). The ncRNA catalogs were finally integrated.

### Gene Families

To understand the adaptive mechanisms of *M. rotundata*, the key gene families associated with environmental adaptation were annotated. For vision‐related genes, the protein sequences of genes related to eye development, differentiation, and phototransduction in fruit fly were first downloaded.^[^ ^26^ ^]^ For chemoreception genes, protein sequences of OR, GR, IR, and OBP in *B. terrestris* were obtained from previous studies.^[^ ^76^ ^]^ For GPCR and detoxification enzyme genes (CCEs, GSTs), protein sequences in representative insect species were downloaded from both Flybase (http://www.flybase.org/) and the NCBI database. For immune‐related genes, seed protein sequences of members involved in Toll and Imd pathways relied on a recently comprehensive study in *Bombyx mori* were obtained.^[^ ^38^ ^]^ Each seed protein sequence was queried using BLASTP (*E*‐value < 1e‐5) in the leafcutter bee OGS and other selected insects. Then, the candidate sequences were aligned again in these OGSs to eliminate omissions by same method above. Finally, the target sequences were extracted and aligned for Pfam domain confirmation in the InterPro database (https://www.ebi.ac.uk/interpro/). Additionally, to orthology analyze the relationships of OR genes, according to the previous method,^[^ ^26^ ^]^ a ML phylogenetic tree of *M. rotundata* and *B. terrestris OR* proteins was inferred using RAxML.

### Molecular Identification of *Toll* Genes

To identify Toll receptors in *M. rotundata*, the hidden Markov model for the Toll family (PF13676) was downloaded from the Pfam database. Toll protein sequences from selected arthropods were used as seed and aligned using MAFFT v7.520. Candidate sequences were then used to create an inner hidden Markov model for further *Toll* gene identification. *Toll* gene orthogroups identified by OrthoFinder v2.5.5 were integrated with other candidate genes to establish the final candidate menu. To analyze the phylogenetic relationships of these *Toll* genes, according to the previous method,^[^ ^42^ ^]^ the ML phylogenetic tree of *M. rotundata* and other arthropod Toll proteins was inferred using RAxML. The MroToll family members were renamed based on their distinct phylogenetic relationships. The 22 MroTolls clustered on the same branch with *D. melanogaster Toll‐1* were named as *MroToll‐1.1*–*MroToll‐1.22*, respectively. The *Mro03.638*, *Mro03.687*, *Mro02.218*, and *Mro02.618* were renamed as *MroTollo*, *MroToll‐6*, *MroToll‐7*, and *MroToll‐9*, respectively. Moreover, two *MroTolls* (*Mro04.412* and *Mro06.106*) belonged to the same clade with *Toll‐7* and *Toll‐8* in the spider *Trichonephila clavipes*. Since *Toll‐7* already had a dedicated evolutionary branch, these two *MroTolls* were collectively named the *MroToll‐8* group.

### Transcriptome Sequencing and Analysis

Transcriptome sequencing was primarily conducted using the illumina sequencing platform. The raw sequencing data were filtered using fastp software. For gene expression analysis, the clean data were aligned to *M. rotundata* genome assembly using HISAT2 v2.1.0. Subsequently, the transcript reads of each gene were counted using htseq‐count from the Python package HTSeq^[^ ^77^ ^]^ and then normalized to reads per kilobase per million mapped reads values. Finally, the differential expression genes were obtained using Bioconductor package edgeR with false discovery rate (FDR) < 0.05 and fold change (FC) > 1.5.

### Phylogenomic Analysis

Protein‐coding sequences of *M. rotundata* and other five hymenopteran species (*O. lignaria* (GCF_012274295.1), *O. bicornis* (GCF_907164935.1), *A. mellifera* (GCF_003254395.2), *B. terrestris* (inner genome), and *N. vitripennis* (GCF_009193385.2)) were used for phylogenomic analysis. Orthologue groups among these six species were identified using OrthoFinder v2.5.5.^[^ ^78^ ^]^ Only the longest transcript was received for analysis of orthologs. A ML phylogenetic tree was then constructed based on valid single‐copy genes. Multiple alignments of protein sequences for each ortholog group were performed using MAFFT v7.520. The alignment results were then automatic aligned and trimmed. Subsequently, RAxML v8.2.12^[^ ^79^ ^]^ was used to construct a ML phylogenetic tree under the best mixed model “PROT + GAMMA + IJTTF” with 500 rapid bootstrap inferences. Divergence time was then inferred using MCMCtree program in Maximum Likelihood (PAML).^[^ ^80^ ^]^ Several calibration time points were designated: 77–85 Mya for *A. mellifera* and *B. terrestris* (TimeTree website, http://www.timetree.org/), and 175–185 Mya for *A. mellifera* and *N. vitripennis*.^[^ ^81^ ^]^ Gene family contraction and expansion analyses were then performed using CAFÉ v5.0,^[^ ^82^ ^]^ with a significance criterion of *p* < 0.05. GO and KEGG enrichment analyses of expanded genes were conducted by OmicShare.

### Genomic Synteny Analysis

Synteny analyses among *M. rotundata*, *O. bicornis*, *A. mellifera*, *B. terrestris*, and *N. vitripennis* were performed using the versatile Python‐based JCVI library.^[^ ^83^ ^]^ The *jcvi.compara.catalog ortholog* with the parameters “‐n 5” and “‐dist 35” were employed to identify syntenic blocks among the genomes. Sequence alignments were primarily conducted using LAST.^[^ ^84^ ^]^ The *jcvi.compara.synteny screen* (‐minspan 30 ‐simple) was then used to process the anchor files and obtained simplified versions of syntenic blocks. Visualized plots were illustrated using *jcvi.graphics.dotplot* and *jcvi.graphics.karyotype*.

### RNA Extraction and qPCR Analysis

Total RNA was extracted from larvae and prepupae using TRIzol reagent (Invitrogen). RNA quality and quantity were assessed via ND‐1000 spectrophotometer (NanoDrop) and 1% agarose gel electrophoresis, respectively. Reverse transcription was carried out using M‐MLV reverse transcriptase (catalogue no. M1701, Promega Corporation). Target gene mRNA levels were quantified using SYBR Green 1 Master Mix (catalogue no. 04707516001, Roche) on a LightCycler 480 (Roche), with RPS18 serving as the internal reference. Dissociation curves were examined to ensure specific amplification for each gene. The qPCR primers are listed in Table S8 (Supporting Information).

### Gene Expression Pattern Analysis between Diapausing and Nondiapausing Prepupae

To validate the expression patterns of Toll pathway genes, the 5th instar larvae and prepupae in both diapause and nondiapause states were collected, immediately frozen in liquid nitrogen, and stored at −80 °C. Based on the observations, individuals collected from the same paper straw, produced by a single female, exhibited similar diapause state, supporting the maternal effect of diapause regulation in this species. Consequently, a “reverse reasoning method” was developed to determine the diapause state of individual prepupae. Specifically, three individuals were collected for the experiment, while the remaining individuals from the same paper straw (>2) were continuously cultured at 30 °C. The diapause states of the test prepupae were assessed by monitoring the developmental progress of their siblings for one month after the prepupal stage. Each group comprised five independent biological replicates, with two individuals per replicate. RNA extraction and qPCR analysis were conducted as described above.

### Expression Pattern Analysis in Response to Bacteria Component Stimulation

To examine the expression patterns of Toll pathway gene induced by microorganisms, diapausing and nondiapausing prepupae were injected with *E. coli* (≈10^6^ cells µL^−1^) or *S. aureus* (≈10^6^ cells µL^−1^). Bacteria cultures were grown overnight in Luria–Bertani broth at 37 °C, then centrifuged at 3000 rpm for 10 min. The cells were washed once with PBS and resuspended in 2 mL PBS. Colony‐forming units were quantified using a serial dilution method. The bacteria were diluted to 7 × 10^6^ cells µL^−1^ and heated at 65 °C for 30 min. Inactivated bacterial cells (140 nL) were microinjected into the lateral side of prepupae abdomen. PBS‐injected *M. rotundata* prepupae served as negative controls. Experimental individuals were collected 6, 12, and 24 h postinjection. Diapause or nondiapause states were determined using the “reverse reasoning method” as described above. Each group included five independent biological replicates, with two individuals per replicate.

### Survival Rate Analysis of Prepupae in Response to Bacteria Challenge

The number of dead individuals was recorded daily for up to ten days postinjection. The detailed counts of dead diapause and nondiapause individuals were determined using the “reverse reasoning method.” Survival curves were generated using GraphPad Prism 6 software, and differences in survival rates were analyzed with the log‐rank (Mantel–Cox) test. At least 20 individuals were included in each treatment group to assess survival rates.

### Statistical Analysis

All data were shown as means ± standard error. The one‐way analysis of variance and Tukey's multiple range tests were used to evaluate the difference among multigroup. Two‐tailed unpaired student's *t*‐test was used for two‐group comparisons. Survival rates were analyzed with the log‐rank (Mantel–Cox) test. Differences were considered statistically significant at *p* < 0.05.

## Conflict of Interest

The authors declare no conflict of interest.

## Author Contributions

X.W. conceived the project. R.S., L.H., and X.W. designed the study. P.D. and L.H. prepared the sequencing samples. R.S. assembled the genome and generated the gene set. P.D., H.H., and L.H. performed experiments. R.S.,P.D., M.Z., R.Z., Z.Z., X.N., and L.H. collected the data. R.S., H.H. and L.H. analyzed the data. R.S., L.H., and X.W. wrote the paper.

## Supporting information



Supporting Information

## Data Availability

The data used in this study have been submitted to the BioProject of the Genome Sequence Archive, ngdc.cncb.ac.cn/bioproject/(accession no. PRGCA037541). In addition, the data can also be obtained from inner leafcutter bee omics database ALCBmine (http://159.226.67.243/viroblast_alcb/alcb.php).
