# Supplementary material for: Chromosome‐Level Genome Assembly of the Leafcutter Bee Megachile rotundata Reveals Its Ecological Adaptation and Pollination Biology
Source: Adv Sci (Weinh). 2025 Mar 26;12(23):2417054. doi: 10.1002/advs.202417054 (PMC12199319; doi:10.1002/advs.202417054)
Supplement: Supplementary file 1 — Supporting Information [file ADVS-12-2417054-s001.docx]

# Supporting Information

Supplementary Material: Contains Figures S1-S14, Table S1-S8.


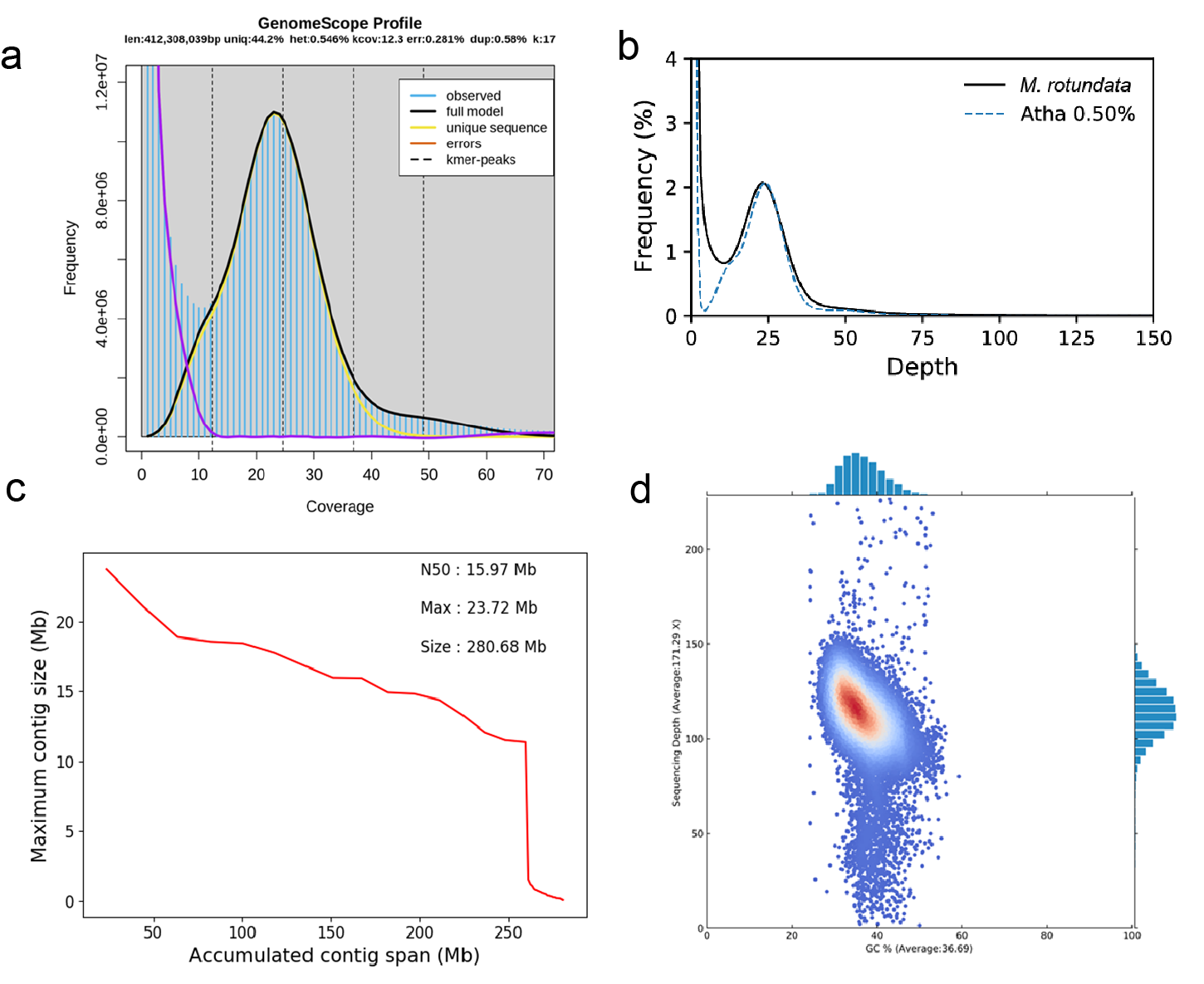


**Fig. S1 Comprehensive information on genome sequencing and assembly.** (a) Genome survey results performed by GenomeScope to estimate *M. rotundata* genome size. (b) *K-mer* distribution curve and heterozygosity simulation for *M. rotundata* genome. The horizontal axis represents *K-mer* depth, while the vertical axis represents *K-mer* depth frequency. (c) Cumulative contig length plot of *M. rotundata* genome. (d) The distribution plot of *M. rotundata* genomic GC content.


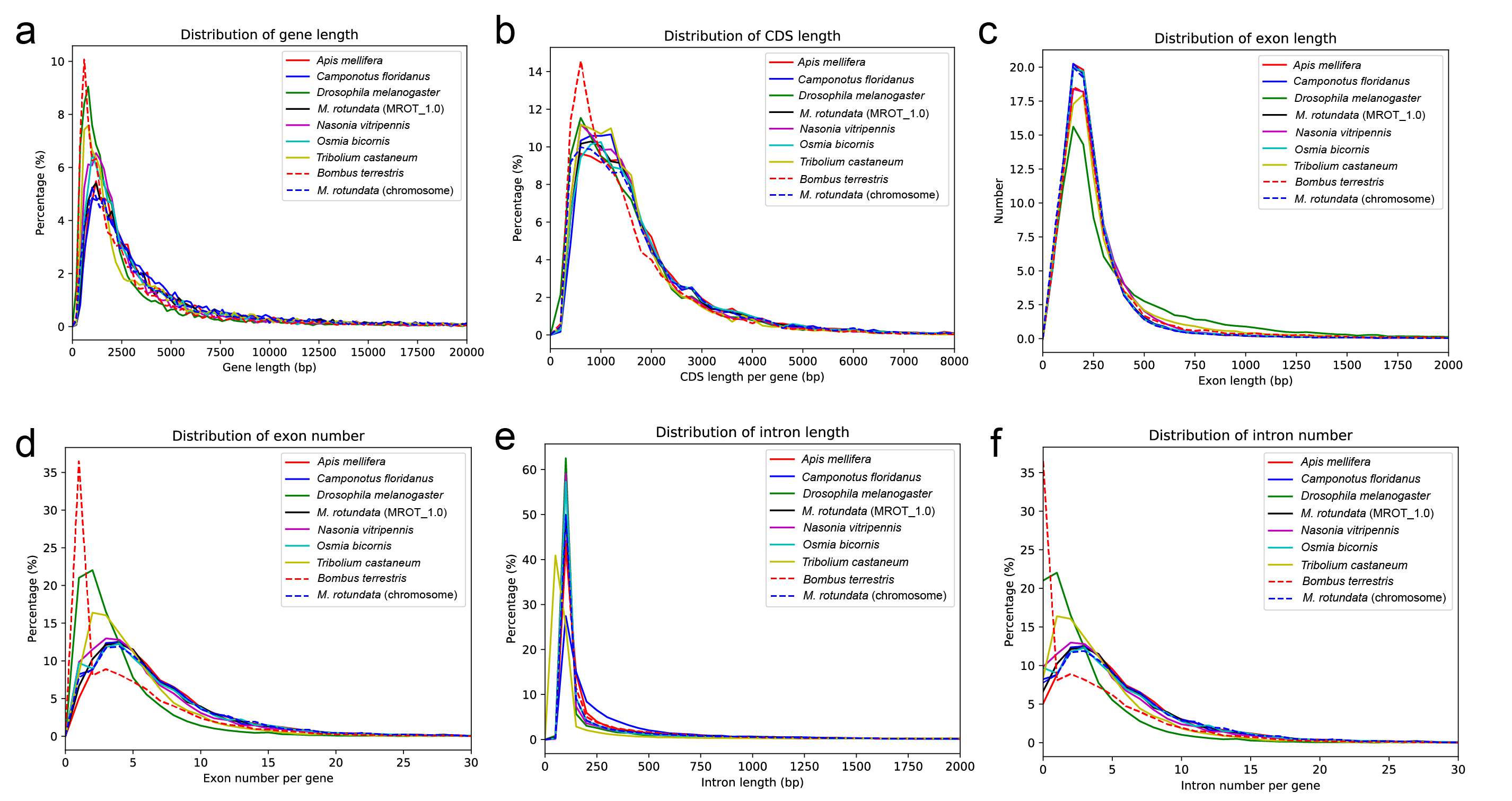


**Fig. S2 The distribution statistics of gene elements in *M. rotundata* and other selected insects.**

~~
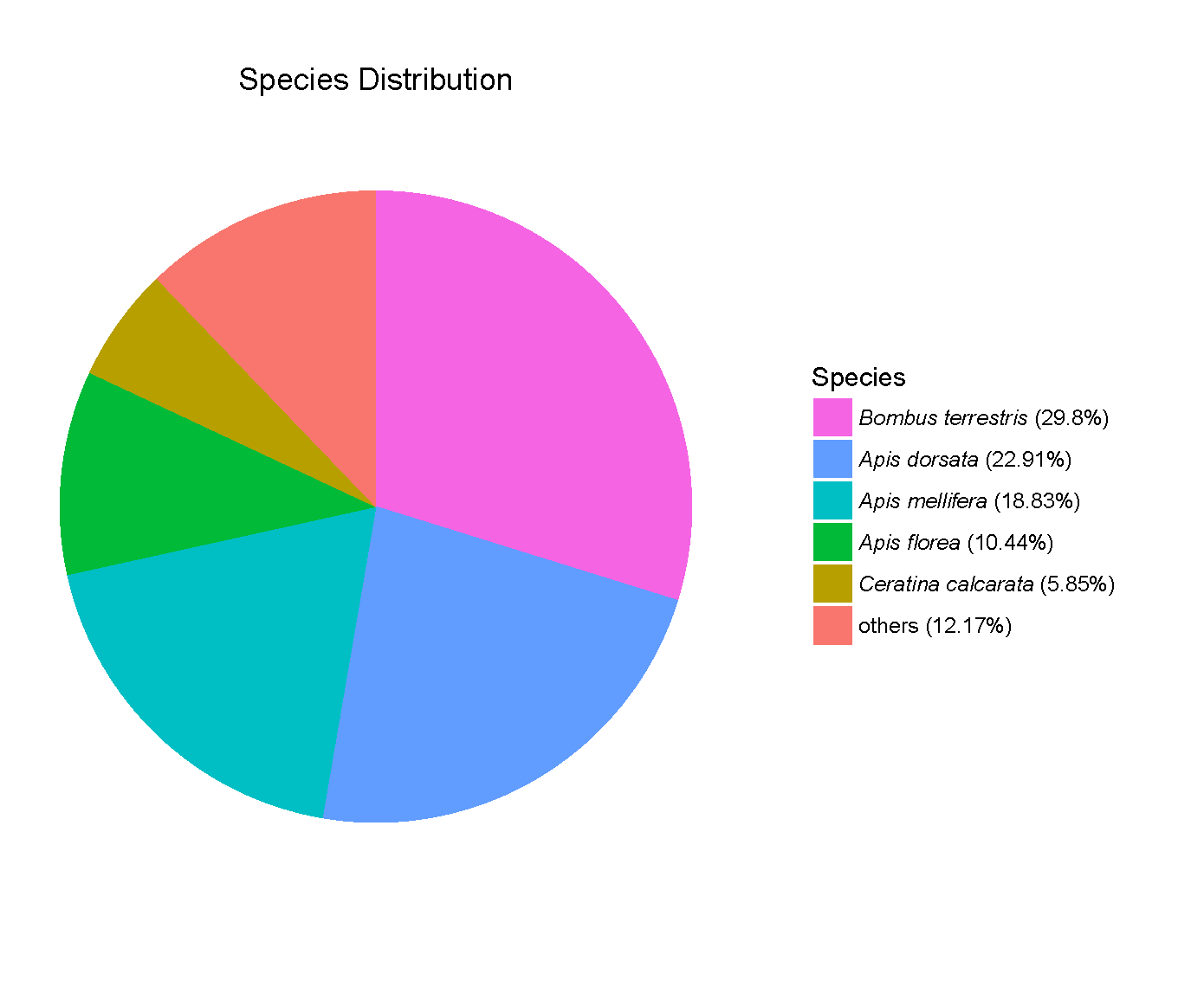
~~

**Fig. S3 *M. rotundata* OGS annotation results corresponding to the species classification chart in the NR database.**


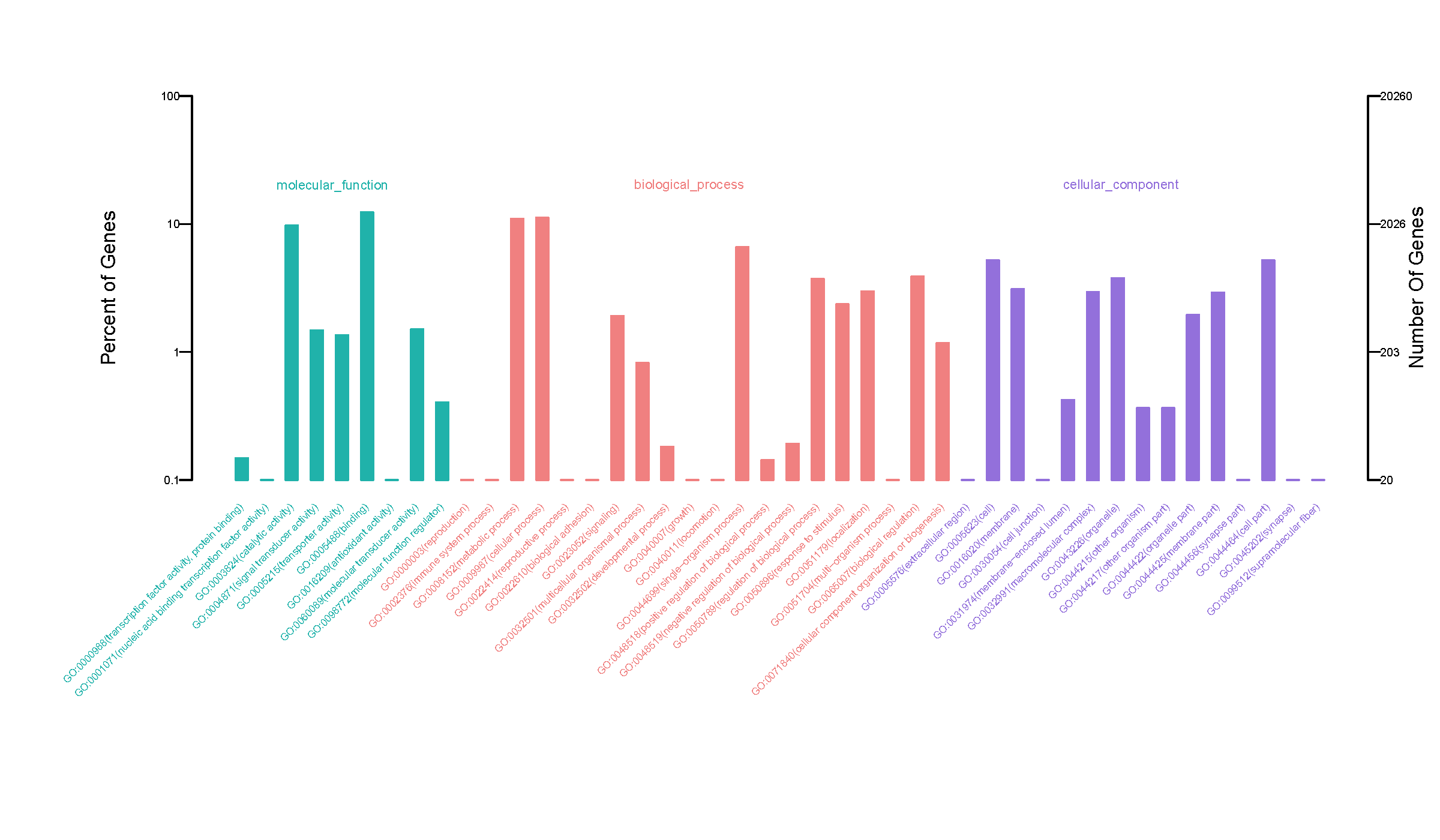


**Fig. S4 Statistical bar chart of GO terms for *M. rotundata* OGS.**


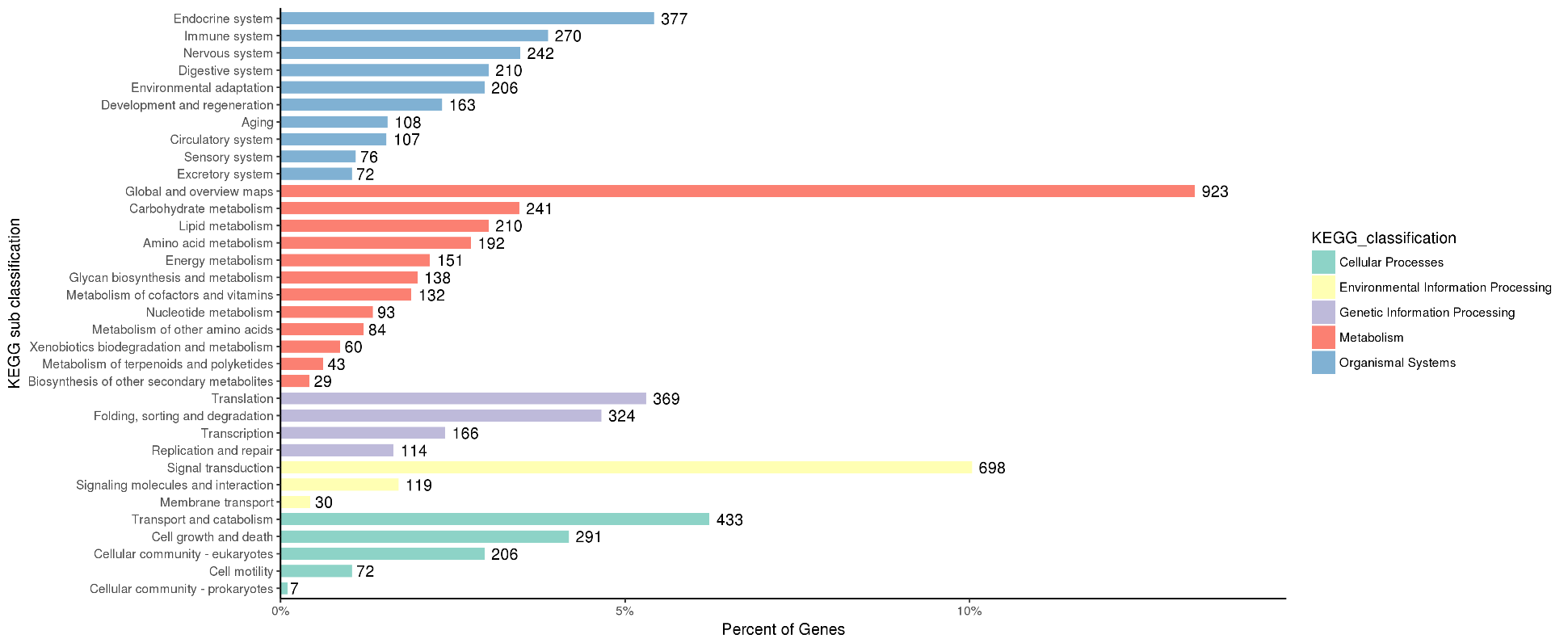


**Fig. S5 KEGG signaling pathway annotation results for the OGS of *M. rotundata*.**


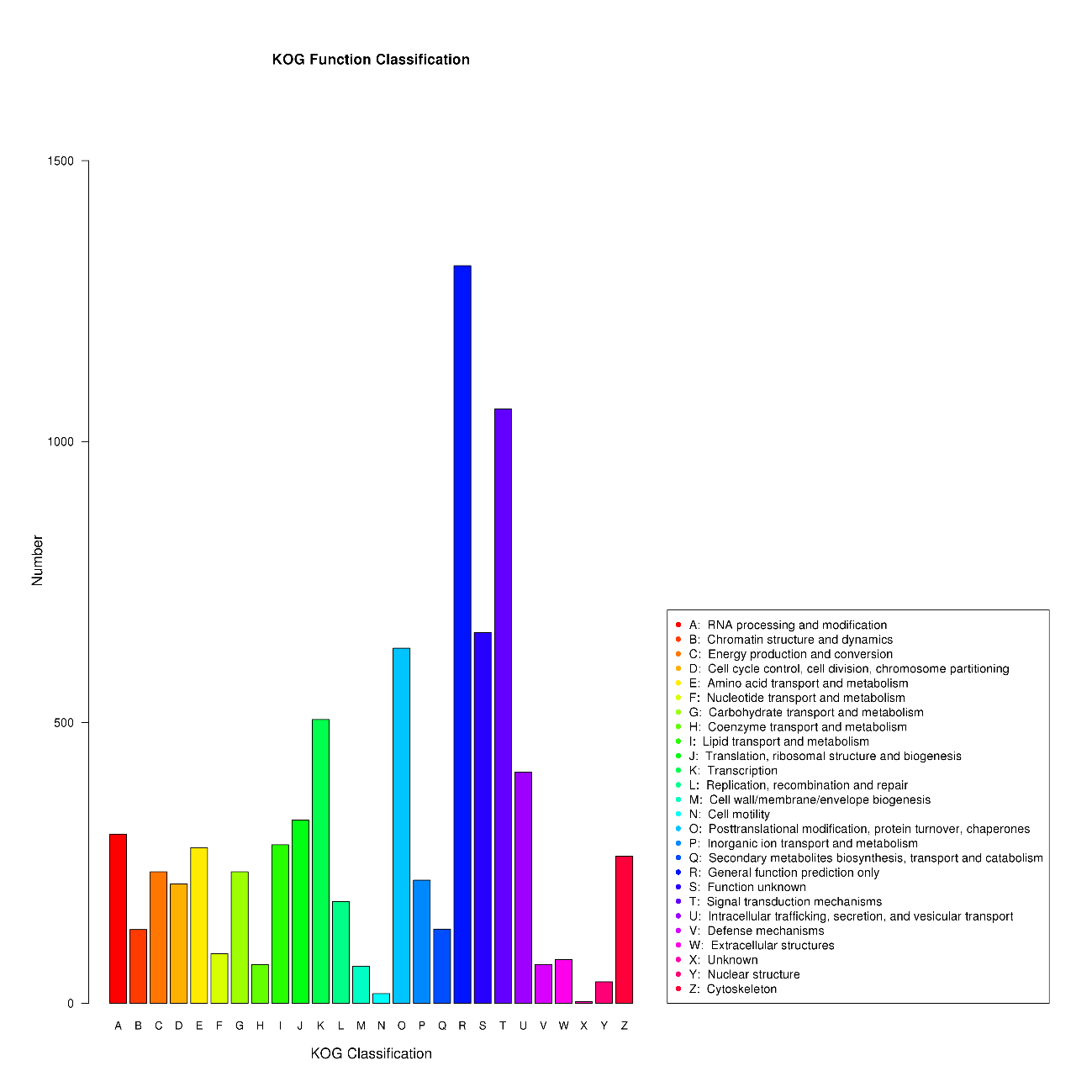


**Fig. S6 Classification chart of KOG annotation results for *M. rotundata* OGS.**


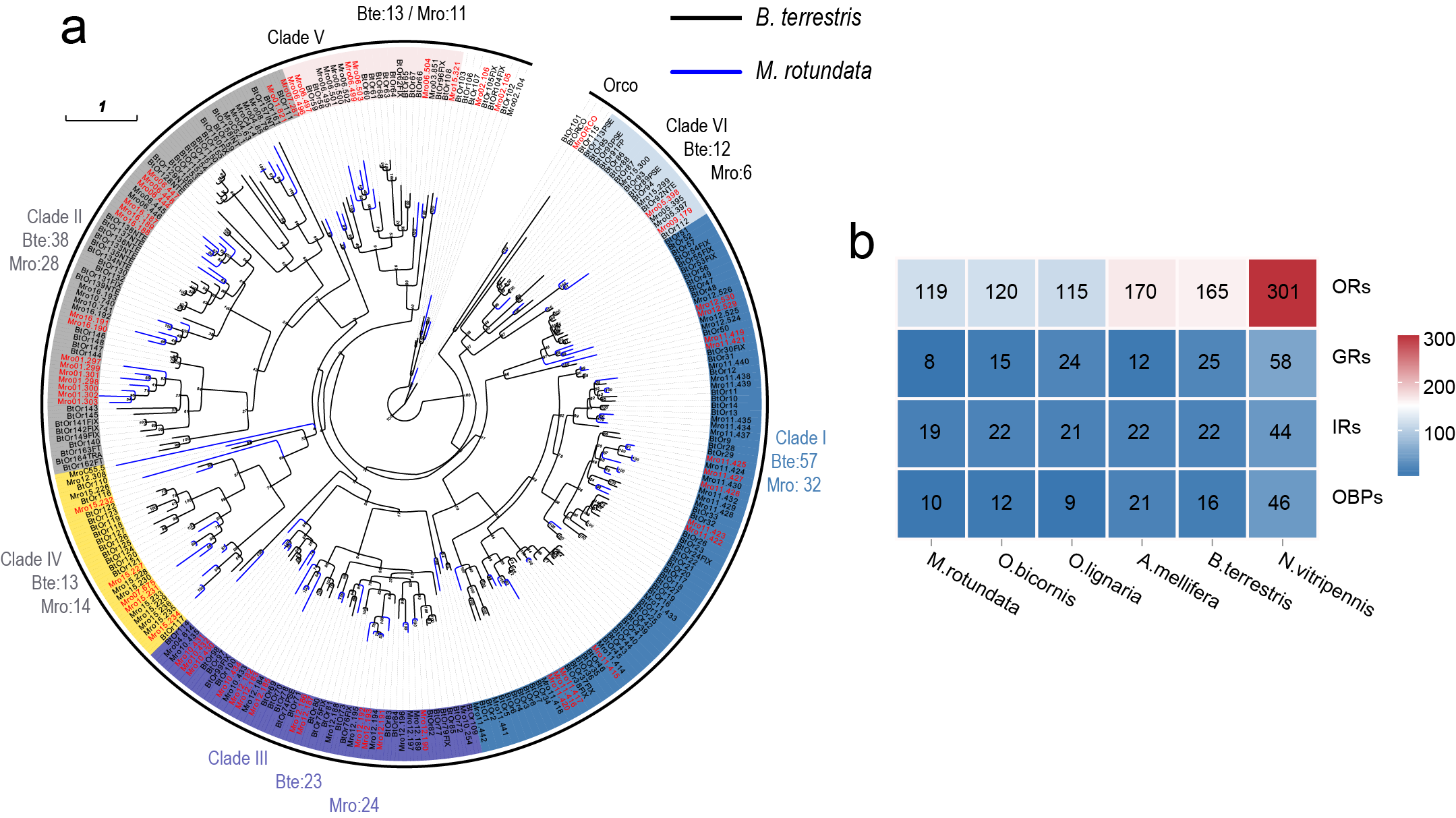


**Fig. S7 Orthology analysis of chemosensory-related genes in *M. rotundata* and other selected hymenopteran species.** (a) Phylogenetic relationship of OR genes from *M. rotundata* and *Bombus terrestris*. Different background colors represent distinct OR subfamilies (I–VI), with geneIDs highlighted in red indicating those highly expressed in *M. rotundata* adult males. (b) Statistics on the number of chemosensory-related genes in *M. rotundata* and other selected hymenopteran species.


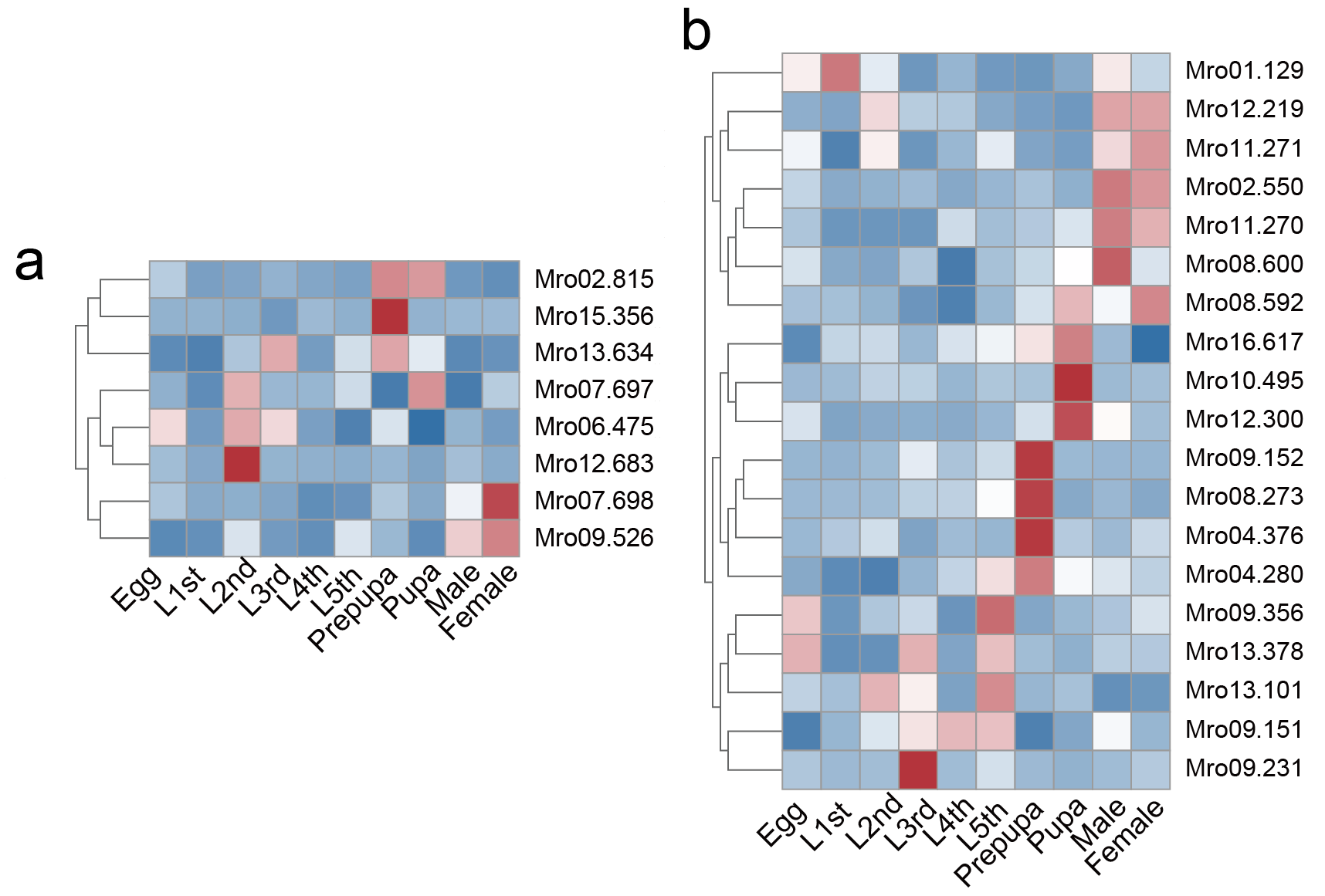


**Fig. S8** **Expression patterns of chemoreception-related genes in *M. rotundata*.** Developmental expression levels of (a) gustatory receptor genes (*GRs*), and (b) ionotropic receptor genes (*IRs*).


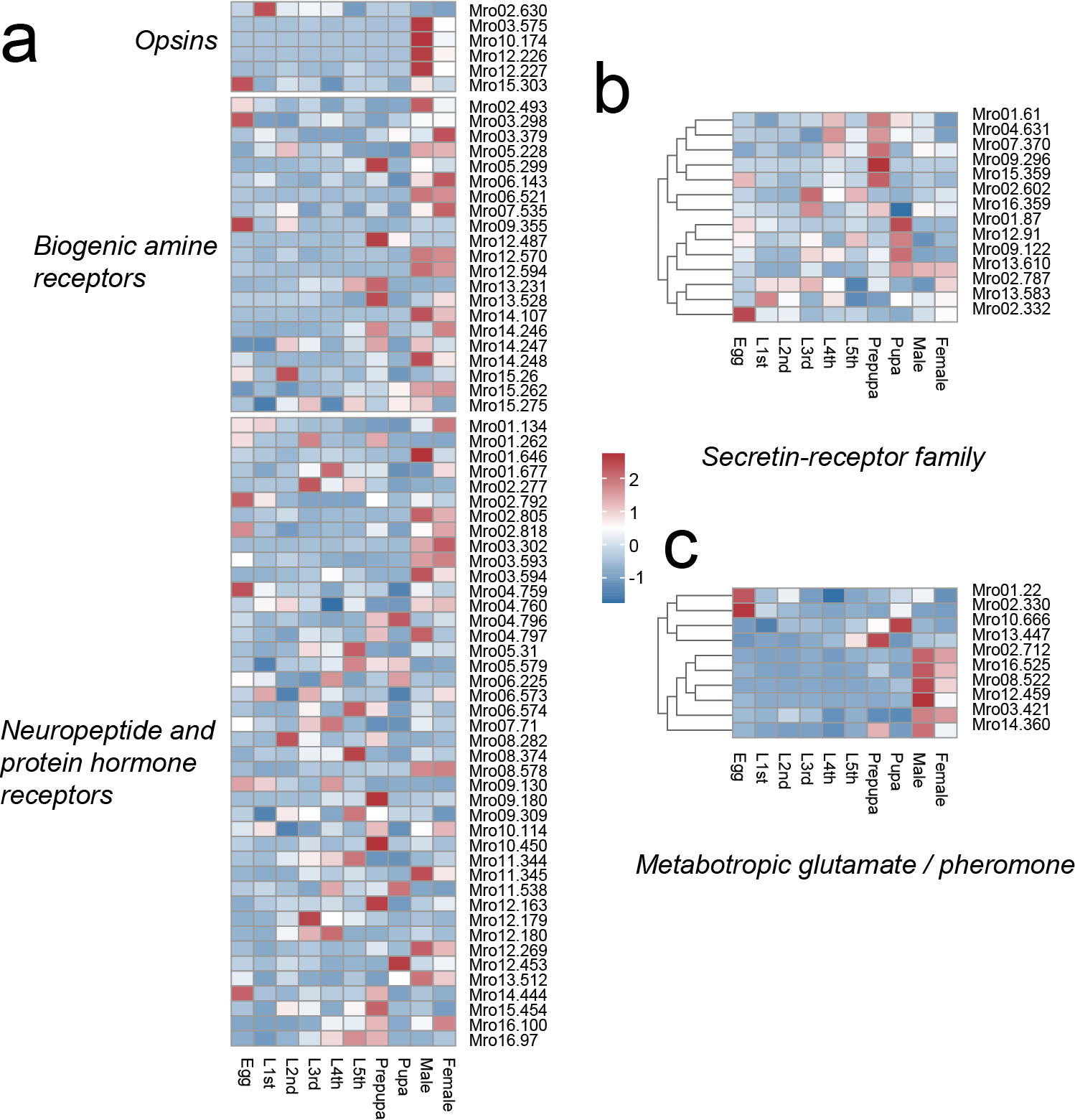


**Fig. S9 Expression patterns of GPCRs in *M. rotundata*.** Developmental expression levels of (a) rhodopsin-like family (*Family-1*) genes, (b) secretin-receptor family (*Family-2*) genes, and (c) metabotropic glutamate/pheromone family (*Family-3*) genes.


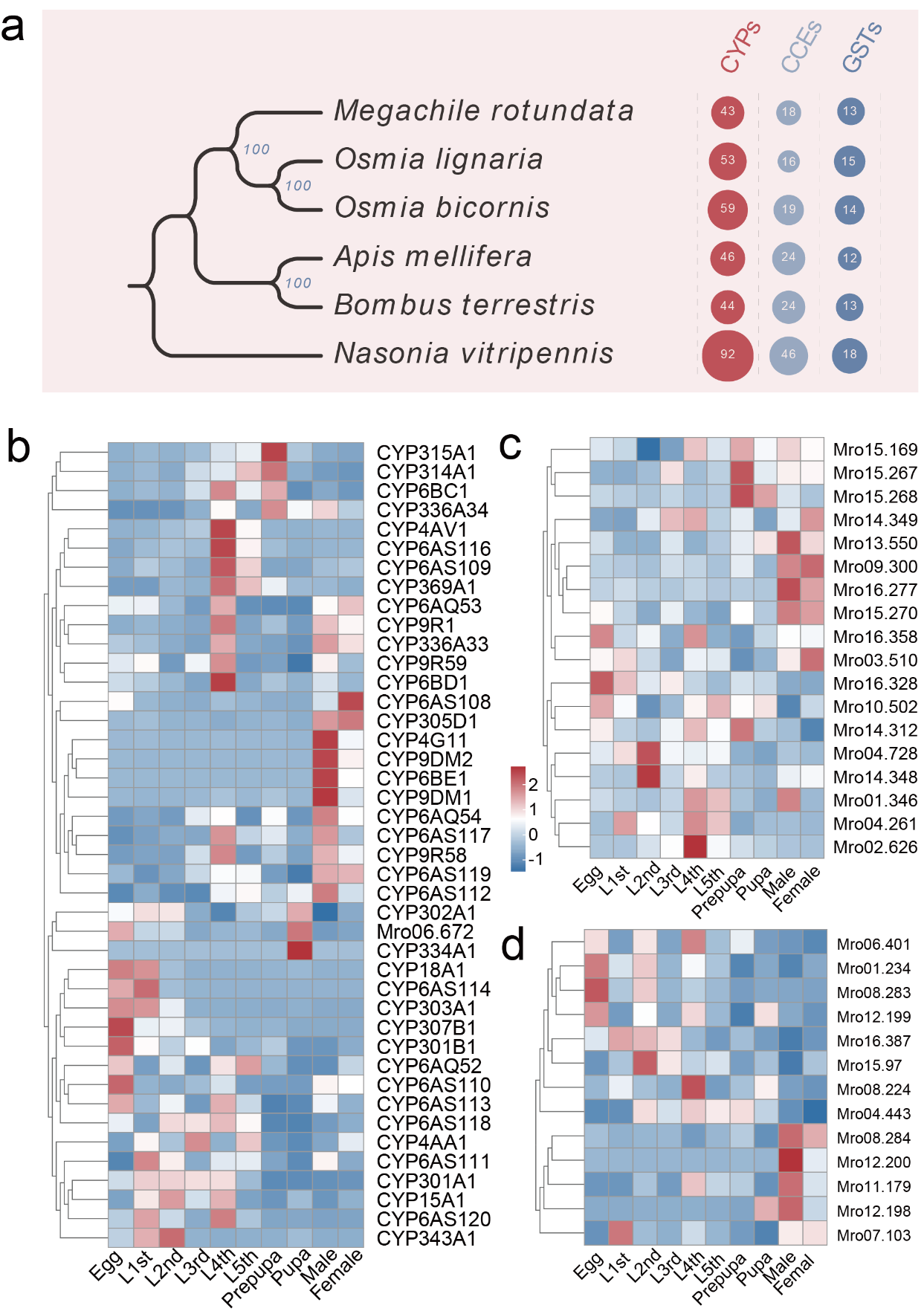


**Fig. S10 Expression patterns of detoxification-related genes in *M. rotundata*.** (a) The number of genes for three detoxification enzymes in *M. rotundata* and other hymenopteran species. Developmental expression patterns of (b) *CYP* genes, (c) Carboxyl/cholinesterases (CCEs) encoding genes, and (d) Glutathione-S-transferases (GSTs) encoding genes.


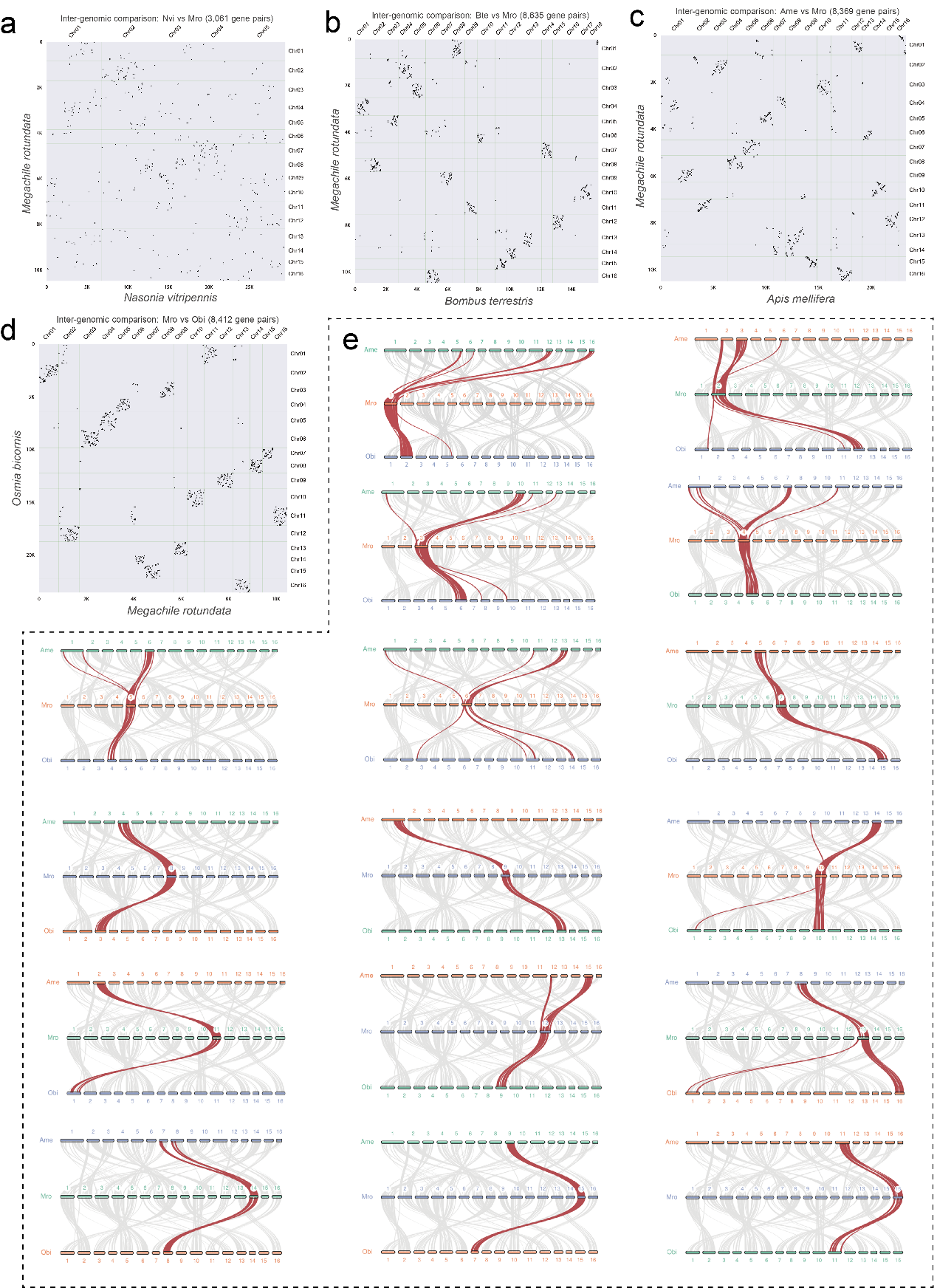


**Fig. S11** **Genomic synteny analysis of *M. rotundata* and other hymenopteran species.** (a) A dot plot representing the syntenic blocks between *N. vitripennis* and *M. rotundata*. (b) The syntenic blocks between *B. terrestris* and *M. rotundata.* (c) A dot plot of *A. mellifera* and *M. rotundata* syntenic relationship*.* (d) A syntenic dot plot of *O. bicornis* and *M. rotundata.* (e) The syntenic correspondence of sixteen chromosomes for *A. mellifera* (*Ame*), *M. rotundata* (*Mro*), and *O. bicornis* (*Obi*), respectively.


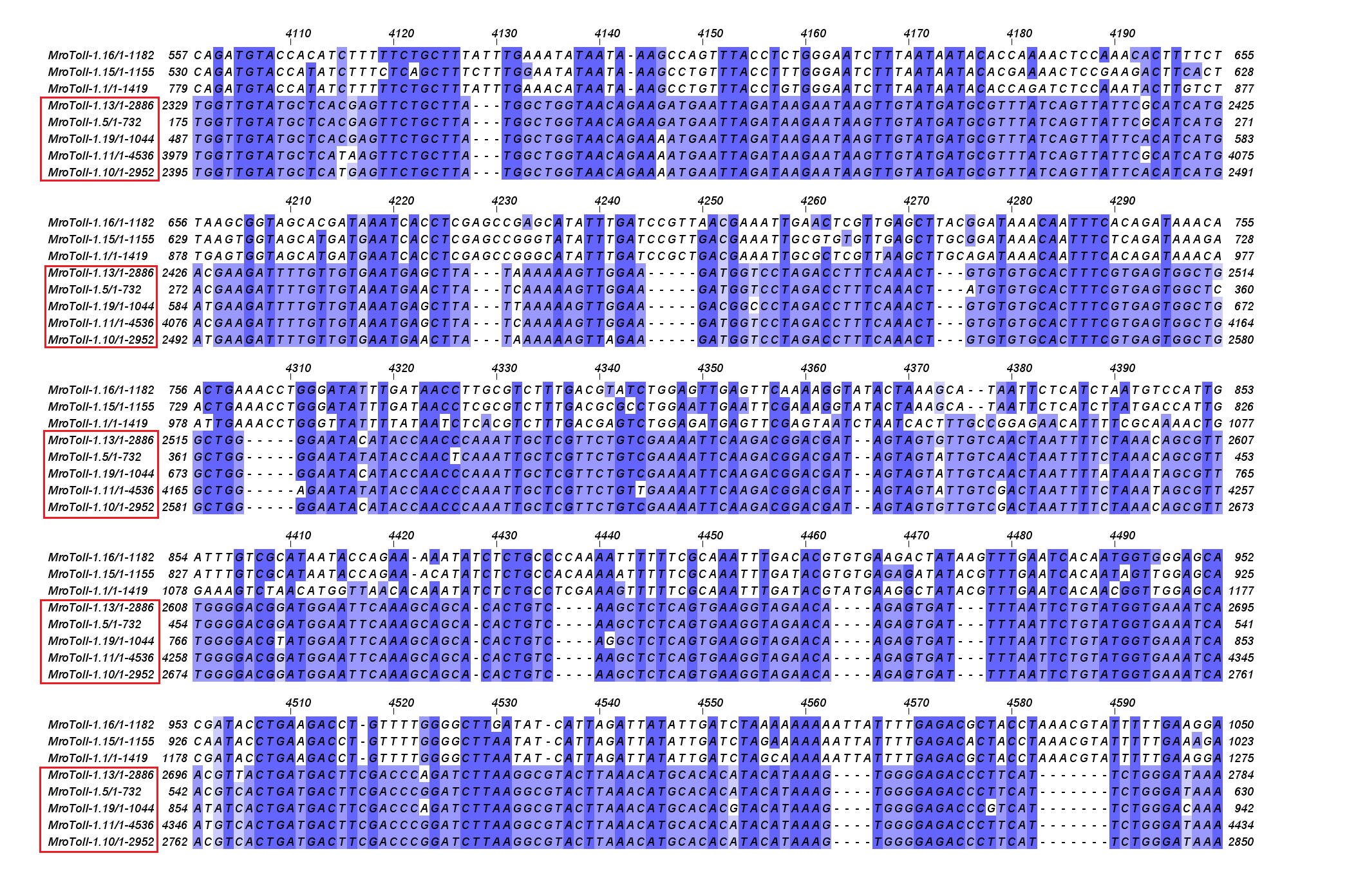


**Fig. S12 Multiple sequence alignments of eight *MroToll-1* subfamily genes that significantly highly expressed in diapausing prepupae.** These eight *MroToll-1* subfamily genes revealed high identity among *MroToll-1.1*, *MroToll-1.15*, and *MroToll-1.16* (*MroToll-1* group1), while *MroToll-1.5*, *MroToll-1.10*, *MroToll-1.11*, *MroToll-1.13*, and *MroToll-1.19* (*MroToll-1* group2, red box) shared highly conserved sequences.


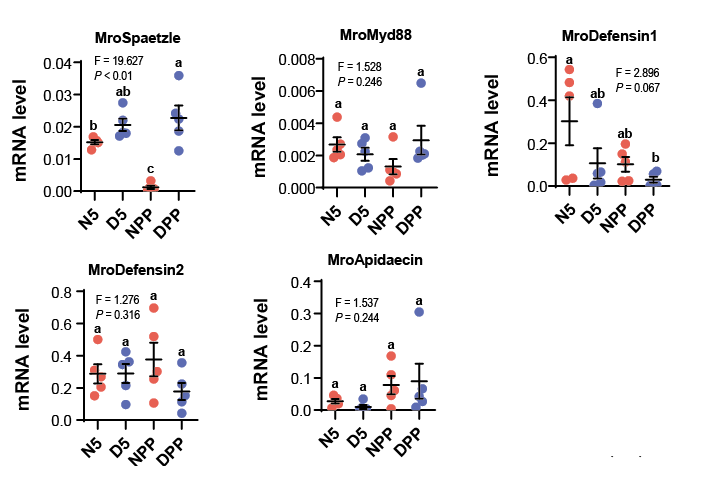


**Fig. S13 The qPCR assay results of key Toll signaling pathway genes in diapause and non-diapause *M. rotundata* individuals at 5th instar larvae and prepupal stage.**


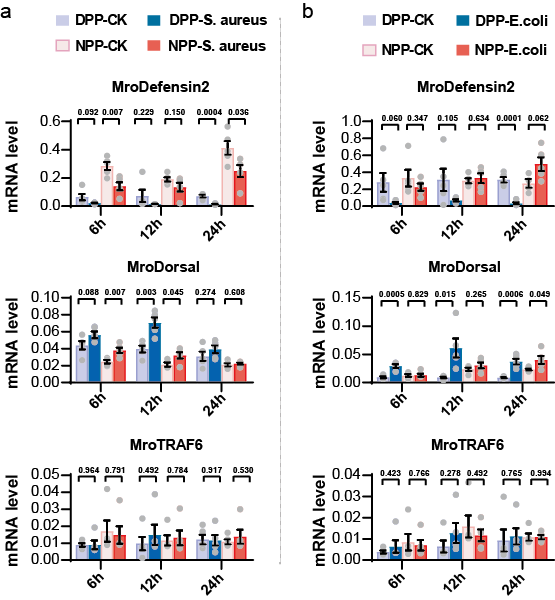


**Fig. S14 Expression pattern analysis of Toll signaling pathway genes in response to bacteria component stimulation.** (a) The mRNA levels of key Toll signaling pathway genes in diapausing (D) or non-diapausing (N) prepupae (PP) upon infection with *S. aureus*. (b) The mRNA levels of key Toll signaling pathway genes in diapausing or non-diapausing prepupae upon infection with *E. coli*.

**Table S1. Preliminary genome assembly results based on BUSCO prediction statistics.**

| **Type** | **Number** | **Percent (%)** |
| --- | --- | --- |
| Complete BUSCOs (C) | 1,357 | 99.27 |
| Complete and single-copy BUSCOs (S) | 1,356 | 99.20 |
| Complete and duplicated BUSCOs (D) | 1 | 0.07 |
| Fragmented BUSCOs (F) | 1 | 0.07 |
| Missing BUSCOs (M) | 9 | 0.66 |
| Total BUSCO groups searched | 1,367 | 100.00 |

Complete BUSCOs (C): The sequence was completely aligned with BUSCO;

Complete and single copy BUSCOs (S): aligning one BUSCO to the next gene;

Complete and duplicated BUSCOs (D): aligning multiple genes with one BUSCO;

Fragmented BUSCOs (F): Partial sequence alignment with BUSCO;

Missing BUSCOs (M): not compared to BUSCO;

Total BUSCO groups searched: Total BUSCO sets.

**Table S2. The CEGMA evaluation and statistics of preliminary genome assembly results.**

| **Type** | **Complete** | | **Complete + Partial** | |
| --- | --- | --- | --- | --- |
|  | **Prots** | **%Completeness** | **Prots** | **%Completeness** |
| Total | 243 | 97.98 | 245 | 98.79 |
| Group1 | 64 | 96.97 | 66 | 100.0 |
| Group2 | 55 | 98.21 | 55 | 98.21 |
| Group3 | 60 | 98.36 | 60 | 98.36 |
| Group4 | 64 | 98.46 | 64 | 98.46 |

Type: type name;

Complete: more than 70% of the genes were assembled;

Complete + partial: The situation of complete comparison and partial comparison;

Prots: number of assembled core genes;

%Completion: The proportion of assembled core genes in the core gene library.

**Table S3. Statistical table of sequence alignment information of the genomes in NT database.**

| **Type** | **Contig number** | **Contig number ratio (%)** | **Contig length (bp)** | **Contig length ratio (%)** |
| --- | --- | --- | --- | --- |
| Metazoa | 33 | 40.74 | 266,181,944 | 94.83 |
| Nohit | 48 | 59.26 | 14,500,306 | 5.17 |
| Total | 81 | 100.00 | 280,682,250 | 100.00 |

Type: Species category information;

Contig Number: The number of Contig;

Contig Number ratio (%): The proportion of Contig numbers;

Contig Length (bp): The sequence length of Contig;

Contig Length ratio (%): The proportion of Contig sequence length to genome size.

**Table S4. Statistics of Hi-C assisted assembly results.**

| **Type** | **Contig length (bp)** | **Contig number** | **Scaffold length (bp)** | **Scaffold number** | **Gap length (bp)** | **Gap number** |
| --- | --- | --- | --- | --- | --- | --- |
| N50 | 15,966,425 | 8 | 16,800,000 | 8 | 100 | 4 |
| N60 | 14,913,090 | 10 | 16,278,735 | 9 | 100 | 5 |
| N70 | 14,365,770 | 12 | 14,819,632 | 11 | 100 | 6 |
| N80 | 12,060,445 | 14 | 13,256,807 | 13 | 100 | 7 |
| N90 | 11,375,586 | 16 | 11,375,586 | 16 | 100 | 8 |
| Longest | 23,723,823 | 1 | 23,723,823 | 1 | 100 | 8 |
| Total | 280,682,250 | 87 | 280,683,050 | 79 | 800 | 8 |
| Length>=1kb | 280,682,250 | 87 | 280,683,050 | 79 | 0 | 0 |
| Length>=2kb | 280,682,250 | 87 | 280,683,050 | 79 | 0 | 0 |
| Length>=5kb | 280,682,250 | 87 | 280,683,050 | 79 | 0 | 0 |

Contig Length: The length of a genome sequence that is continuous and does not contain N;

Contig Number: The number of consecutive sequences in the genome that do not contain N;

Scaffold Length: Genome Scaffold sequence length;

Scaffold Number: The number of genomic scaffold sequences;

Gap Length: The length of N consecutive genomes;

Gap Number: The number of consecutive N in the genome;

N50: Genome N50;

N60: The added length reaches 60% of the total Contig length, and the last added Contig length;

N70: The added length reaches 70% of the total Contig length, and the last added Contig length;

N80: The added length reaches 80% of the total Contig length, and the last added Contig length;

N90: The added length reaches 90% of the total Contig length, and the last added Contig length;

Longest: the longest Contig length in the genome assembly sequence;

Total: The length of all sequences in the genome;

Length>=1kb: a sequence with a length greater than or equal to 1Kb;

Length>=2kb: a sequence with a length greater than or equal to 2Kb;

Length>=5kb: A sequence with a length greater than or equal to 5kb.

**Table S5. The statistical results of various repetitive sequences in *M. rotundata* genome.**

| **Class** | **Order** | **Super family** | **Number of elements** | **Length of sequence (bp)** | **Percentage of sequence (%)** |
| --- | --- | --- | --- | --- | --- |
| **Class I** |  |  | 93,820 | 32,348,394 | 11.52 |
|  | SINE |  | 2,642 | 3,417,314 | 1.22 |
|  |  | Unknown | 2,607 | 3,415,053 | 1.22 |
|  |  | Other | 35 | 2,261 | 0.00 |
|  | LTR |  | 60,660 | 21,489,904 | 7.66 |
|  |  | Unknown | 42,922 | 14,638,380 | 5.22 |
|  |  | Copia | 2,399 | 1,134,026 | 0.40 |
|  |  | Pao | 4,731 | 1,595,620 | 0.57 |
|  |  | Gypsy | 9,361 | 4,044,729 | 1.44 |
|  |  | Other | 1,247 | 77,149 | 0.03 |
|  | LINE |  | 30,518 | 7,441,176 | 2.65 |
|  |  | Unknown | 14,927 | 2,975,958 | 1.06 |
|  |  | R1 | 3,247 | 1,530,234 | 0.55 |
|  |  | R2 | 836 | 349,994 | 0.12 |
|  |  | I | 2,356 | 921,687 | 0.33 |
|  |  | I-Jockey | 1,204 | 372,991 | 0.13 |
|  |  | L2 | 2,984 | 558,773 | 0.20 |
|  |  | Other | 4,964 | 731,539 | 0.26 |
| **Class II** |  |  | 157,266 | 34,309,181 | 12.22 |
|  | DNA |  | 144,519 | 30,420,963 | 10.84 |
|  |  | Unknown | 114,428 | 25,306,467 | 9.02 |
|  |  | TcMar-Tc1 | 6,058 | 1,254,062 | 0.45 |
|  |  | PiggyBac | 1,889 | 499,551 | 0.18 |
|  |  | TcMar-Tc4 | 1,581 | 414,196 | 0.15 |
|  |  | TcMar-Mariner | 2,740 | 672,327 | 0.24 |
|  |  | Other | 17,823 | 2,274,360 | 0.81 |
|  | MITE |  | 9,415 | 3,211,774 | 1.14 |
|  |  | Unknown | 9,415 | 3,211,774 | 1.14 |
|  | RC |  | 3,332 | 676,444 | 0.24 |
|  |  | Helitron | 3,332 | 676,444 | 0.24 |
| **Total TEs** |  |  | 251,086 | 66,657,575 | 23.75 |
| **Tandem Repeats** |  |  | 60,051 | 9,818,014 | 3.50 |
|  | Tandem repeat |  | 47,668 | 9,664,395 | 3.44 |
|  | STR |  | 12,383 | 153,619 | 0.05 |
| **Unknown** |  |  | 53,557 | 13,645,524 | 4.86 |
| **Simple repeats** |  |  | 4,592 | 995,514 | 0.35 |
| **Low complexity** |  |  | 216 | 39,583 | 0.01 |
| **Other** |  |  | 3,831 | 969,905 | 0.35 |
| **Total Repeats** |  |  | 373,333 | 92,126,115 | 32.82 |

**Note**: Other in the table refers to sequences classified by annotations but not belonging to the above categories, while Unknown refers to sequences that annotations cannot classify.

**Table S6. ncRNAs analysis in *M. rotundata* genome.**

| **Type** | **Number** | **Average length (bp)** | **Total length (bp)** | **Percentage (%)** |
| --- | --- | --- | --- | --- |
| regulatory | 110 | 48.05 | 5,285 | 0.0019 |
| tRNA | 206 | 76.29 | 15,716 | 0.0056 |
| ncRNA | 117 | 132.24 | 15,472 | 0.0055 |
| rRNA | 163 | 1,614.82 | 263,215 | 0.0938 |
| **rRNA** | | | | |
| 18S | 32 | 2,196.16 | 70,277 | 0.025 |
| 28S | 39 | 4,393.56 | 171,349 | 0.061 |
| 5S | 54 | 113.81 | 6,146 | 0.0022 |
| 5.8S | 35.00 | 155.17 | 5,431 | 0.0019 |
| **ncRNA** | | | | |
| snRNA | 7.00 | 117.71 | 824 | 0.0003 |
| miRNA | 48.00 | 82.5 | 3,960 | 0.0014 |
| spliceosomal | 52 | 164.9 | 8,575 | 0.0031 |

**Table S7. Annotation details of GPCR superfamily genes in *M. rotundata* OGS*.***

| **Length** | ***M. rotundata* ID** | **NR Annotation** | **Swiss-Prot Annotation** |
| --- | --- | --- | --- |
| **Family-1 (Rhodopsin family)** | | | |
| **Opsin** | | | |
| **675** | ***Mro02.630*** | **XP_003394682.1 beta-3 adrenergic receptor [Bombus terrestris]** | **Rhodopsin, GQ-coupled OS=*Mizuhopecten yessoensis* OX=6573 GN=SCOP1 PE=1 SV=1** |
| **329** | ***Mro15.303*** | **XP_003397947.1 green-sensitive opsin [Bombus terrestris]** | **Rhodopsin OS=*Sminthopsis crassicaudata* OX=9301 GN=RHO PE=2 SV=2** |
| **385** | ***Mro12.226*** | **XP_012172197.1 rhodopsin, long-wavelength [Bombus terrestris]** | **Rhodopsin, long-wavelength OS=*Apis mellifera* OX=7460 PE=1 SV=1** |
| **392** | ***Mro12.227*** | **XP_011150409.1 rhodopsin isoform X2 [Harpegnathos saltator]** | **Rhodopsin OS=*Camponotus atriceps* OX=104420 PE=2 SV=1** |
| **380** | ***Mro10.174*** | **XP_003400385.1 opsin, blue-sensitive [Bombus terrestris]** | **Opsin, blue-sensitive OS=*Apis mellifera* OX=7460 GN=BLOP PE=1 SV=2** |
| **371** | ***Mro03.575*** | **XP_003695717.1 PREDICTED: opsin, ultraviolet-sensitive [Apis florea]** | **Opsin, ultraviolet-sensitive OS=*Apis mellifera* OX=7460 GN=UVOP PE=1 SV=1** |
| **Biogenic amine receptors** | | | |
| **399** | ***Mro06.521*** | **XP_003400301.1 5-hydroxytryptamine receptor isoform X1 [Bombus terrestris]** | **5-hydroxytryptamine receptor OS=*Heliothis virescens* OX=7102 PE=2 SV=1** |
| **225** | ***Mro03.379*** | **XP_003398431.1 5-hydroxytryptamine receptor 1A [Bombus terrestris]** | **5-hydroxytryptamine receptor 2A OS=*Macaca mulatta* OX=9544 GN=HTR2A PE=2 SV=1** |
| **405** | ***Mro05.228*** | **XP_003695657.1 PREDICTED: putative tyramine receptor 2 [Apis florea]** | **5-hydroxytryptamine receptor 7 OS=*Xenopus laevis* OX=8355 GN=htr7 PE=2 SV=1** |
| **505** | ***Mro03.298*** | **XP_006609951.1 PREDICTED: 5-hydroxytryptamine receptor 1-like isoform X2 [Apis dorsata]** | **5-hydroxytryptamine receptor 1 OS=*Drosophila melanogaster* OX=7227 GN=5-HT7 PE=2 SV=1** |
| **651** | ***Mro15.262*** | **XP_006561568.2 D2-like dopamine receptor isoform X2 [Apis mellifera]** | **Dopamine D2-like receptor OS=*Drosophila melanogaster* OX=7227 GN=Dop2R PE=2 SV=2** |
| **632** | ***Mro15.275*** | **XP_003397807.1 5-hydroxytryptamine receptor 2A isoform X1 [Bombus terrestris]** | **D(2) dopamine receptor A OS=*Xenopus laevis* OX=8355 GN=drd2-a PE=2 SV=1** |
| **464** | ***Mro12.570*** | **XP_026301042.1 dopamine receptor 2 isoform X1 [Apis mellifera]** | **Dopamine receptor 2 OS=*Drosophila melanogaster* OX=7227 GN=Dop1R2 PE=2 SV=1** |
| **359** | ***Mro12.594*** | **XP_003401071.1 dopamine receptor 1 isoform X2 [*Bombus terrestris*]** | **Dopamine receptor 1 OS=*Drosophila melanogaster* OX=7227 GN=Dop1R1 PE=2 SV=2** |
| **313** | ***Mro05.299*** | **XP_003698435.1 PREDICTED: probable G-protein coupled receptor 52 [*Apis florea*]** | **G-protein coupled receptor 52 OS=*Mus musculus* OX=10090 GN=Gpr52 PE=2 SV=1** |
| **598** | ***Mro13.231*** | **XP_006608645.1 PREDICTED: muscarinic acetylcholine receptor DM1-like [Apis dorsata]** | **Muscarinic acetylcholine receptor DM1 OS=*Drosophila melanogaster* OX=7227 GN=mAChR-A PE=2 SV=2** |
| **917** | ***Mro15.26*** | **XP_003397629.1 probable muscarinic acetylcholine receptor gar-1 [Bombus terrestris]** | **Muscarinic acetylcholine receptor gar-2 OS=Caenorhabditis elegans OX=6239 GN=gar-2 PE=2 SV=3** |
| **428** | ***Mro14.107*** | **XP_397139.3 octopamine receptor beta-1R [Apis mellifera]** | **Octopamine receptor beta-1R OS=Drosophila melanogaster OX=7227 GN=Octbeta1R PE=2 SV=1** |
| **570** | ***Mro14.246*** | **XP_003402376.1 octopamine receptor beta-2R isoform X1 [Bombus terrestris]** | **Octopamine receptor beta-2R OS=Drosophila melanogaster OX=7227 GN=Octbeta2R PE=2 SV=2** |
| **416** | ***Mro14.247*** | **XP_006618041.1 PREDICTED: octopamine receptor beta-3R-like isoform X1 [Apis dorsata]** | **Octopamine receptor beta-1R OS=Drosophila melanogaster OX=7227 GN=Octbeta1R PE=2 SV=1** |
| **361** | ***Mro14.248*** | **XP_006618042.1 PREDICTED: octopamine receptor beta-3R-like isoform X2 [Apis dorsata]** | **Octopamine receptor beta-3R OS=Drosophila melanogaster OX=7227 GN=Octbeta3R PE=2 SV=4** |
| **545** | ***Mro12.487*** | **XP_012172321.1 octopamine receptor Oamb isoform X1 [Bombus terrestris]** | **Probable G-protein coupled receptor No9 OS=Amphibalanus amphitrite OX=1232801 PE=3 SV=1** |
| **402** | ***Mro09.355*** | **XP_003392960.1 putative tyramine receptor 2 isoform X2 [Bombus terrestris]** | **Tyramine receptor 1 OS=Locusta migratoria OX=7004 GN=GCR1 PE=1 SV=1** |
| **1050** | ***Mro07.535*** | **XP_006613628.1 PREDICTED: uncharacterized protein LOC102672484 isoform X1 [Apis dorsata]** | **Adenosine receptor A2b OS=Homo sapiens OX=9606 GN=ADORA2B PE=2 SV=1** |
| **506** | ***Mro02.493*** | **XP_003698754.1 PREDICTED: LOW QUALITY PROTEIN: adenosine receptor A2b-like [Apis florea]** | **Adenosine receptor A2b OS=Oryctolagus cuniculus OX=9986 GN=ADORA2B PE=2 SV=1** |
| **692** | ***Mro06.143*** | **XP_016773287.2 uncharacterized protein LOC100578696 [Apis mellifera]** | **Histamine H1 receptor OS=Rattus norvegicus OX=10116 GN=Hrh1 PE=1 SV=1** |
| **453** | ***Mro13.528*** | **XP_003393279.1 trace amine-associated receptor 9 isoform X1 [Bombus terrestris]** | **Trace amine-associated receptor 1 OS=Rattus norvegicus OX=10116 GN=Taar1 PE=2 SV=1** |
| **Neuropeptide and protein hormone receptors** | | | |
| **408** | ***Mro01.134*** | **XP_003402126.1 cardioacceleratory peptide receptor [Bombus terrestris]** | **Cardioacceleratory peptide receptor OS=Drosophila melanogaster OX=7227 GN=CCAP-R PE=2 SV=4** |
| **448** | ***Mro01.646*** | **XP_003399932.1 RYamide receptor isoform X2 [Bombus terrestris]** | **RYamide receptor OS=Drosophila melanogaster OX=7227 GN=RYa-R PE=2 SV=2** |
| **168** | ***Mro16.97*** | **XP_006608028.1 PREDICTED: neuropeptide Y receptor-like isoform X1 [Apis dorsata]** | **RYamide receptor OS=Drosophila melanogaster OX=7227 GN=RYa-R PE=2 SV=2** |
| **319** | ***Mro16.100*** | **XP_006608028.1 PREDICTED: neuropeptide Y receptor-like isoform X1 [Apis dorsata]** | **RYamide receptor OS=Drosophila melanogaster OX=7227 GN=RYa-R PE=2 SV=2** |
| **375** | ***Mro06.225*** | **XP_012165487.1 RYamide receptor [Bombus terrestris]** | **Melatonin receptor type 1C OS=Xenopus laevis OX=8355 GN=mtnr1c PE=2 SV=1** |
| **461** | ***Mro15.454*** | **XP_003397405.1 prolactin-releasing peptide receptor [Bombus terrestris]** | **Neuropeptide FF receptor 2 OS=Homo sapiens OX=9606 GN=NPFFR2 PE=1 SV=2** |
| **506** | ***Mro08.374*** | **XP_003394933.1 orexin receptor type 1 isoform X2 [Bombus terrestris]** | **Neuropeptide FF receptor 2 OS=Homo sapiens OX=9606 GN=NPFFR2 PE=1 SV=2** |
| **414** | ***Mro08.578*** | **XP_026295776.1 orexin receptor type 2-like [Apis mellifera]** | **Orexin receptor type 2 OS=Mus musculus OX=10090 GN=Hcrtr2 PE=2 SV=2** |
| **536** | ***Mro01.677*** | **XP_006562433.1 cholecystokinin receptor-like [Apis mellifera]** | **Cholecystokinin receptor type A OS=Homo sapiens OX=9606 GN=CCKAR PE=1 SV=1** |
| **421** | ***Mro02.792*** | **XP_003394391.1 somatostatin receptor type 2 [Bombus terrestris]** | **Somatostatin receptor type 4 OS=Rattus norvegicus OX=10116 GN=Sstr4 PE=1 SV=1** |
| **353** | ***Mro10.450*** | **XP_012171985.1 allatostatin-A receptor [Bombus terrestris]** | **Allatostatin-A receptor OS=Bombyx mori OX=7091 GN=AR PE=2 SV=1** |
| **471** | ***Mro06.574*** | **XP_006609591.1 PREDICTED: gastrin-releasing peptide receptor-like isoform X1 [Apis dorsata]** | **Neuropeptide CCHamide-1 receptor OS=Drosophila melanogaster OX=7227 GN=CCHa1-R PE=2 SV=2** |
| **405** | ***Mro06.573*** | **XP_006609698.1 PREDICTED: gastrin-releasing peptide receptor-like [Apis dorsata]** | **Neuropeptide CCHamide-1 receptor OS=Drosophila melanogaster OX=7227 GN=CCHa1-R PE=2 SV=2** |
| **563** | ***Mro08.282*** | **XP_003694328.1 PREDICTED: neuromedin-U receptor 2-like isoform X2 [Apis florea]** | **Pyrokinin-1 receptor OS=Drosophila melanogaster OX=7227 GN=PK1-R PE=2 SV=1** |
| **458** | ***Mro10.114*** | **XP_026675315.1 QRFP-like peptide receptor isoform X1 [Ceratina calcarata]** | **Pyroglutamylated RF-amide peptide receptor OS=Rattus norvegicus OX=10116 GN=Qrfpr PE=2 SV=1** |
| **387** | ***Mro11.345*** | **XP_003393783.1 neuropeptides capa receptor isoform X2 [Bombus terrestris]** | **Neuropeptides capa receptor OS=Drosophila melanogaster OX=7227 GN=CapaR PE=2 SV=3** |
| **456** | ***Mro11.344*** | **XP_026302166.1 capa receptor-like GPCR isoform X1 [Apis mellifera]** | **Neuropeptides capa receptor OS=Drosophila melanogaster OX=7227 GN=CapaR PE=2 SV=3** |
| **419** | ***Mro12.163*** | **XP_006612331.1 PREDICTED: tachykinin-like peptides receptor 99D-like isoform X2 [Apis dorsata]** | **Tachykinin-like peptides receptor 99D OS=Drosophila melanogaster OX=7227 GN=TkR99D PE=2 SV=2** |
| **434** | ***Mro13.512*** | **XP_003398616.1 tachykinin-like peptides receptor 86C isoform X1 [Bombus terrestris]** | **Tachykinin-like peptides receptor 86C OS=Drosophila melanogaster OX=7227 GN=TkR86C PE=2 SV=2** |
| **530** | ***Mro14.444*** | **XP_003691479.1 PREDICTED: neuromedin-U receptor 2-like [Apis florea]** | **Pyrokinin-1 receptor OS=Drosophila melanogaster OX=7227 GN=PK1-R PE=2 SV=1** |
| **449** | ***Mro02.818*** | **XP_003394353.1 FMRFamide receptor [Bombus terrestris]** | **FMRFamide receptor OS=Drosophila melanogaster OX=7227 GN=FMRFaR PE=2 SV=1** |
| **438** | ***Mro12.269*** | **XP_026672567.1 free fatty acid receptor 4-like isoform X2 [Ceratina calcarata]** | **Neuropeptide Y receptor type 2 OS=Mus musculus OX=10090 GN=Npy2r PE=2 SV=2** |
| **249** | ***Mro12.179*** | **XP_003401591.1 neuropeptide SIFamide receptor [Bombus terrestris]** | **Neuropeptide SIFamide receptor OS=Drosophila melanogaster OX=7227 GN=SIFaR PE=2 SV=2** |
| **339** | ***Mro12.180*** | **XP_003401591.1 neuropeptide SIFamide receptor [Bombus terrestris]** | **Neuropeptide SIFamide receptor OS=Drosophila melanogaster OX=7227 GN=SIFaR PE=2 SV=2** |
| **750** | ***Mro09.180*** | **XP_006625154.1 PREDICTED: relaxin receptor 1-like isoform X1 [Apis dorsata]** | **Relaxin receptor 1 OS=Mus musculus OX=10090 GN=Rxfp1 PE=2 SV=1** |
| **414** | ***Mro02.805*** | **XP_012176061.1 probable G-protein coupled receptor B0563.6 [Bombus terrestris]** | **Probable G-protein coupled receptor B0563.6 OS=Caenorhabditis elegans OX=6239 GN=B0563.6 PE=3 SV=2** |
| **384** | ***Mro03.593*** | **XP_003398466.1 G-protein coupled receptor moody isoform X1 [Bombus terrestris]** | **G-protein coupled receptor moody OS=Drosophila melanogaster OX=7227 GN=moody PE=2 SV=2** |
| **538** | ***Mro03.594*** | **XP_017881188.2 G-protein coupled receptor moody isoform X1 [Ceratina calcarata]** | **G-protein coupled receptor moody OS=Drosophila pseudoobscura pseudoobscura OX=46245 GN=moody PE=3 SV=2** |
| **385** | ***Mro04.796*** | **XP_011063453.1 PREDICTED: probable G-protein coupled receptor B0563.6 [Acromyrmex echinatior]** | **Probable G-protein coupled receptor B0563.6 OS=Caenorhabditis elegans OX=6239 GN=B0563.6 PE=3 SV=2** |
| **256** | ***Mro04.797*** | **XP_026300439.1 probable G-protein coupled receptor B0563.6 isoform X1 [Apis mellifera]** | **Probable G-protein coupled receptor B0563.6 OS=Caenorhabditis elegans OX=6239 GN=B0563.6 PE=3 SV=2** |
| **342** | ***Mro11.538*** | **XP_012170122.1 protein trapped in endoderm-1 [Bombus terrestris]** | **Protein trapped in endoderm-1 OS=Drosophila melanogaster OX=7227 GN=Tre1 PE=1 SV=1** |
| **506** | ***Mro12.453*** | **XP_003401590.1 alpha-2B adrenergic receptor [Bombus terrestris]** | **Alpha-2C adrenergic receptor OS=Danio rerio OX=7955 GN=adra2c PE=3 SV=1** |
| **467** | ***Mro05.31*** | **XP_026830555.1 thyrotropin-releasing hormone receptor [Ooceraea biroi]** | **Alpha-2C adrenergic receptor OS=Danio rerio OX=7955 GN=adra2c PE=3 SV=1** |
| **403** | ***Mro09.130*** | **XP_003698007.1 PREDICTED: prostaglandin E2 receptor EP2 subtype isoform X1 [Apis florea]** | **Prostaglandin E2 receptor EP3 subtype OS=Sus scrofa OX=9823 GN=PTGER3 PE=2 SV=1** |
| **348** | ***Mro05.579*** | **XP_003690609.1 PREDICTED: gonadotropin-releasing hormone II receptor [Apis florea]** | **Gonadotropin-releasing hormone II receptor OS=Clarias gariepinus OX=13013 PE=2 SV=2** |
| **237** | ***Mro04.760*** | **EGI63519.1 Thyrotropin-releasing hormone receptor [Acromyrmex echinatior]** | **Thyrotropin-releasing hormone receptor OS=Mus musculus OX=10090 GN=Trhr PE=2 SV=1** |
| **371** | ***Mro04.759*** | **XP_026301815.1 growth hormone secretagogue receptor type 1 isoform X4 [Apis mellifera]** | **Growth hormone secretagogue receptor type 1 OS=Mus musculus OX=10090 GN=Ghsr PE=2 SV=3** |
| **414** | ***Mro03.302*** | **XP_012168758.1 gonadotropin-releasing hormone receptor [Bombus terrestris]** | **Gonadotropin-releasing hormone receptor OS=Octopus vulgaris OX=6645 GN=GNRHR PE=2 SV=1** |
| **1025** | ***Mro01.262*** | **XP_006623299.1 PREDICTED: lutropin-choriogonadotropic hormone receptor-like isoform X1 [Apis dorsata]** | **Lutropin-choriogonadotropic hormone receptor OS=Mus musculus OX=10090 GN=Lhcgr PE=2 SV=1** |
| **462** | ***Mro09.309*** | **XP_026824017.1 probable G-protein coupled receptor 139 [Ooceraea biroi]** | **-** |
| **429** | ***Mro07.71*** | **XP_006614027.1 PREDICTED: probable G-protein coupled receptor 139-like [Apis dorsata]** | **Sex peptide receptor OS=Drosophila melanogaster OX=7227 GN=SPR PE=1 SV=1** |
| **392** | ***Mro02.277*** | **XP_003402026.1 sex peptide receptor [Bombus terrestris]** | **Sex peptide receptor OS=Drosophila melanogaster OX=7227 GN=SPR PE=1 SV=1** |
| **Family-2 (secretin-receptor family)** | | | |
| **493** | ***Mro15.359*** | **XP_026669652.1 parathyroid hormone 2 receptor [*Ceratina calcarata*]** | **Secretin receptor OS=Rattus norvegicus OX=10116 GN=Sctr PE=1 SV=1** |
| **477** | ***Mro02.602*** | **XP_006608505.1 PREDICTED: diuretic hormone receptor-like isoform X1 [*Apis dorsata*]** | **Diuretic hormone receptor OS=Acheta domesticus OX=6997 PE=2 SV=1** |
| **367** | ***Mro16.359*** | **XP_006623055.1 PREDICTED: calcitonin gene-related peptide type 1 receptor-like isoform X1 [Apis dorsata]** | **Calcitonin receptor OS=Cavia porcellus OX=10141 GN=CALCR PE=2 SV=1** |
| **581** | ***Mro13.583*** | **XP_003403217.1 parathyroid hormone/parathyroid hormone-related peptide receptor isoform X3 [Bombus terrestris]** | **Parathyroid hormone/parathyroid hormone-related peptide receptor OS=*Sus scrofa* OX=9823 GN=PTH1R PE=1 SV=1** |
| **1301** | ***Mro09.122*** | **XP_006616500.1 PREDICTED: latrophilin Cirl-like isoform X5 [Apis dorsata]** | **Latrophilin Cirl OS=*Drosophila ananassae* OX=7217 GN=Cirl PE=3 SV=1** |
| **325** | ***Mro01.61*** | **XP_011066794.1 PREDICTED: cadherin EGF LAG seven-pass G-type receptor 1-like [Acromyrmex echinatior]** | **Cadherin EGF LAG seven-pass G-type receptor 3 OS=*Mus musculus* OX=10090 GN=Celsr3 PE=2 SV=2** |
| **3167** | ***Mro01.87*** | **XP_026674249.1 protocadherin-like wing polarity protein stan isoform X2 [Ceratina calcarata]** | **Protocadherin-like wing polarity protein stan OS=*Drosophila melanogaster* OX=7227 GN=stan PE=1 SV=4** |
| **498** | ***Mro02.332*** | **XP_006614236.1 PREDICTED: G-protein coupled receptor Mth2-like [Apis dorsata]** | **G-protein coupled receptor Mth2 OS=*Drosophila yakuba* OX=7245 GN=mth2 PE=3 SV=1** |
| **244** | ***Mro02.787*** | **XP_003394252.1 LOW QUALITY PROTEIN: probable G-protein coupled receptor Mth-like 1 [Bombus terrestris]** | **Probable G-protein coupled receptor Mth-like 1 OS=*Drosophila melanogaster* OX=7227 GN=mthl1 PE=2 SV=1** |
| **540** | ***Mro04.631*** | **XP_001120657.3 G-protein coupled receptor Mth2 isoform X2 [Apis mellifera]** | **G-protein coupled receptor Mth2 OS=*Drosophila simulans* OX=7240 GN=mth2 PE=3 SV=1** |
| **427** | ***Mro07.370*** | **XP_003696223.1 PREDICTED: G-protein coupled receptor Mth2-like [Apis florea]** | **G-protein coupled receptor Mth2 OS=*Drosophila yakuba* OX=7245 GN=mth2 PE=3 SV=1** |
| **726** | ***Mro09.296*** | **XP_026675575.1 G-protein coupled receptor Mth2-like [Ceratina calcarata]** | **G-protein coupled receptor Mth2 OS=*Drosophila simulans* OX=7240 GN=mth2 PE=3 SV=1** |
| **1556** | ***Mro12.91*** | **XP_006610210.1 PREDICTED: probable G-protein coupled receptor 125-like [Apis dorsata]** | **Adhesion G protein-coupled receptor A3 OS=*Mus musculus* OX=10090 GN=Adgra3 PE=2 SV=3** |
| **463** | ***Mro13.610*** | **XP_003692545.1 PREDICTED: PDF receptor-like isoform X2 [Apis florea]** | **PDF receptor OS=*Drosophila melanogaster* OX=7227 GN=Pdfr PE=1 SV=2** |
| **Family-3 (metabotropic glutamate/pheromone)** | | | |
| **897** | ***Mro01.22*** | **XP_026667943.1 LOW QUALITY PROTEIN: metabotropic glutamate receptor 6-like [Ceratina calcarata]** | **Metabotropic glutamate receptor OS=*Drosophila melanogaster* OX=7227 GN=mGluR PE=1 SV=2** |
| **909** | ***Mro16.525*** | **XP_003399281.1 metabotropic glutamate receptor [Bombus terrestris]** | **Metabotropic glutamate receptor OS=*Drosophila melanogaster* OX=7227 GN=mGluR PE=1 SV=2** |
| **1022** | ***Mro10.666*** | **XP_003400418.1 metabotropic glutamate receptor 8 [Bombus terrestris]** | **Metabotropic glutamate receptor 7 OS=*Homo sapiens* OX=9606 GN=GRM7 PE=1 SV=1** |
| **458** | ***Mro02.330*** | **XP_003394160.1 uncharacterized protein LOC100646941 [Bombus terrestris]** | **-** |
| **814** | ***Mro03.421*** | **XP_006616838.1 PREDICTED: probable G-protein coupled receptor CG31760-like isoform X5 [Apis dorsata]** | **Probable G-protein coupled receptor CG31760 OS=*Drosophila melanogaster* OX=7227 GN=CG31760 PE=1 SV=3** |
| **1645** | ***Mro08.522*** | **XP_003402432.1 uncharacterized protein LOC100643790 isoform X3 [Bombus terrestris]** | **Gamma-aminobutyric acid type B receptor subunit 2 OS=*Homo sapiens* OX=9606 GN=GABBR2 PE=1 SV=1** |
| **821** | ***Mro02.712*** | **XP_003394329.1 gamma-aminobutyric acid type B receptor subunit 1 isoform X4 [Bombus terrestris]** | **Gamma-aminobutyric acid type B receptor subunit 1 OS=*Homo sapiens* OX=9606 GN=GABBR1 PE=1 SV=1** |
| **1343** | ***Mro14.360*** | **XP_020719959.1 gamma-aminobutyric acid type B receptor subunit 2 [Bombus terrestris]** | **Gamma-aminobutyric acid type B receptor subunit 2 OS=*Homo sapiens* OX=9606 GN=GABBR2 PE=1 SV=1** |
| **857** | ***Mro12.459*** | **XP_003401363.1 protein bride of sevenless isoform X1 [Bombus terrestris]** | **Protein bride of sevenless OS=*Drosophila melanogaster* OX=7227 GN=boss PE=2 SV=3** |
| **1078** | ***Mro13.447*** | **XP_016769061.2 probable G-protein coupled receptor 158 [Apis mellifera]** | **Probable G-protein coupled receptor 158 OS=*Rattus norvegicus* OX=10116 GN=Gpr158 PE=1 SV=1** |

**Table S8. Primers for qPCR experiments.**

| **Genes** | **Forward primer**  **(5’-3’)** | **Reverse primer**  **(5’-3’)** |
| --- | --- | --- |
| ***MroToll-1* (*Group-1*)** | TCAAGACGGACGATAGTAG | CTCTTGTTCTACCTTCACTG |
| ***MroToll-1* (*Group-2*)** | ACTTGTTCAACTTATTCCTGAA | TTCAAATGGTTTCTGACGATGG |
| ***MroDefensin-2*** | CTCCAACTCATTGTCGTCAGC | TCCATCATAAACCGCCTCT |
| ***MroDefensin-1*** | AGTCGTCGGAGACAGTGCTT | GGTCCTTGAAGTTGGTTTTGC |
| ***MroDorsal*** | ATGGAGGCTCAGTGGGTA | TAGATGACTGCTCGTTGG |
| ***MroTRAF6*** | ACAGTATGATGAGTGACCC | CAATCCAATCCGTCATCG |
| ***MroMyD88*** | TTGGCAAGAAAGGCAGAAGG | TTCTATGACTTCAGCCGA |
| ***MroSpaetzle*** | CCTGTAGAAGAGAGGATAG | ATAACCCTCGGCAAGACCT |
| ***MroApidaecin*** | TCTAATCCACCAAACTGTC | ACGCTGTTGCGATAATTCC |
| ***MroRPS18*** | ACGAATTGGCAAGATGTCGCTCGT | GCATAACGGCGACCAACACC |
